# Technology-Based Compensation Assessment and Detection of Upper Extremity Activities of Stroke Survivors: Systematic Review

**DOI:** 10.2196/34307

**Published:** 2022-06-13

**Authors:** Xiaoyi Wang, Yan Fu, Bing Ye, Jessica Babineau, Yong Ding, Alex Mihailidis

**Affiliations:** 1 School of Mechanical Science and Engineering, Huazhong University of Science and Technology Wuhan China; 2 KITE - Toronto Rehabilitation Institute, University Health Network Toronto, ON Canada; 3 Department of Occupational Science and Occupational Therapy, University of Toronto Toronto, ON Canada; 4 Library and Information Services, University Health Network Toronto, ON Canada; 5 Department of Rehabilitation Medicine, Hubei Provincial Hospital of Traditional Chinese Medicine Wuhan China

**Keywords:** stroke, upper extremity rehabilitation, UE rehabilitation, compensation, assessment, technology, sensor, artificial intelligence, AI

## Abstract

**Background:**

Upper extremity (UE) impairment affects up to 80% of stroke survivors and accounts for most of the rehabilitation after discharge from the hospital release. Compensation, commonly used by stroke survivors during UE rehabilitation, is applied to adapt to the loss of motor function and may impede the rehabilitation process in the long term and lead to new orthopedic problems. Intensive monitoring of compensatory movements is critical for improving the functional outcomes during rehabilitation.

**Objective:**

This review analyzes how technology-based methods have been applied to assess and detect compensation during stroke UE rehabilitation.

**Methods:**

We conducted a wide database search. All studies were independently screened by 2 reviewers (XW and YF), with a third reviewer (BY) involved in resolving discrepancies. The final included studies were rated according to their level of clinical evidence based on their correlation with clinical scales (with the same tasks or the same evaluation criteria). One reviewer (XW) extracted data on publication, demographic information, compensation types, sensors used for compensation assessment, compensation measurements, and statistical or artificial intelligence methods. Accuracy was checked by another reviewer (YF). Four research questions were presented. For each question, the data were synthesized and tabulated, and a descriptive summary of the findings was provided. The data were synthesized and tabulated based on each research question.

**Results:**

A total of 72 studies were included in this review. In all, 2 types of compensation were identified: disuse of the affected upper limb and awkward use of the affected upper limb to adjust for limited strength, mobility, and motor control. Various models and quantitative measurements have been proposed to characterize compensation. Body-worn technology (25/72, 35% studies) was the most used sensor technology to assess compensation, followed by marker-based motion capture system (24/72, 33% studies) and marker-free vision sensor technology (16/72, 22% studies). Most studies (56/72, 78% studies) used statistical methods for compensation assessment, whereas heterogeneous machine learning algorithms (15/72, 21% studies) were also applied for automatic detection of compensatory movements and postures.

**Conclusions:**

This systematic review provides insights for future research on technology-based compensation assessment and detection in stroke UE rehabilitation. Technology-based compensation assessment and detection have the capacity to augment rehabilitation independent of the constant care of therapists. The drawbacks of each sensor in compensation assessment and detection are discussed, and future research could focus on methods to overcome these disadvantages. It is advised that open data together with multilabel classification algorithms or deep learning algorithms could benefit from automatic real time compensation detection. It is also recommended that technology-based compensation predictions be explored.

## Introduction

### Background

Stroke occurs almost every 2 seconds worldwide, affecting 13.7 million people each year [1]. Approximately 80% of stroke survivors are affected by upper extremity (UE) motor impairment, and 50% have UE motor dysfunction even 4 years after stroke [2]. Poststroke UE rehabilitation plays an important role in UE motor function recovery. Current research has shown that 2 competing mechanisms may occur simultaneously during the UE function recovery process: motor recovery and compensation. Motor recovery is defined as the “reappearance of elemental motor patterns presents prior to central nervous system injury,” whereas compensation is defined as “the appearance of new motor patterns resulting from the adaptation of remaining motor elements or substitution” [3]. Common compensatory strategies include excessive trunk displacement during reaching movement [3]. Recent research argues that the frequent use of compensation may lead to long-term chronic pain in overused joints, limited function in the impaired muscles, suboptimal motor recovery in the impaired arm, and an abnormal UE movement pattern in activities of daily living [3-5]. Therefore, timely detection and appropriate correction of compensation are important The mechanism underlying UE rehabilitation is neuroplasticity, which refers to the rewiring or reorganization of the brain by creating new connections between brain cells after a stroke [6]. More specifically, to realize brain plasticity, extensive, intensive, task-oriented UE movement repetitions must be performed [7]. Traditionally, UE rehabilitation is completed in a hospital under the supervision of a therapist, in which case some compensatory behaviors can be avoided or corrected under the guidance of the therapist [8]. However, not all compensation can be observed in a timely manner by therapists [9]. Moreover, the UE rehabilitation protocol is labor-intensive for therapists, and there are not enough skilled therapists to support such huge demands [10]. Technology-based therapies, such as robot-assisted therapy and virtual reality (VR) therapy [11], have been used to facilitate UE rehabilitation after stroke in recent years. However, an important prerequisite for taking full advantage of these technology-based therapies is that stroke survivors can correctly perform the therapy exercises as intended, which means that compensation should be automatically detected and corrected in these therapy systems [12]. Technologies could provide more fine-grained automatic compensation monitoring in less-supervised UE therapies so that stroke survivors could continue with the required exercises independent of therapists. Despite the recent increase in attention given to technology for automatic compensation assessment and detection, no systematic reviews have been conducted in this area.

### Objectives

The main goal of this review was to explore how technology-based methods were used to assess and detect compensation without the constant care of therapists.

Our research questions (RQs) are as follows:

1. What models are used to assess and detect compensation in poststroke UE activities?
2. What measurements are used to evaluate compensatory movements?
3. What types of sensor technology are used for compensation assessment and detection?
4. Which statistical or artificial intelligence (AI) methods are used for compensation assessment and detection?

## Methods

The systematic review was performed according to PRISMA (Preferred Reporting Items for Systematic Reviews and Meta-Analyses) guidelines (Multimedia Appendix 1).

### Information Sources and Search Strategy

A comprehensive search strategy was developed and executed by an information specialist (JB). The search strategy was originally developed in MEDLINE ALL (Ovid), in consultation with the research team. The search results were then translated into other databases and study registries. The following electronic databases were searched: MEDLINE (R) ALL (Ovid), Embase and Embase Classic (Ovid), Cochrane Central Register of Controlled Trials (CENTRAL, Ovid), Health Technology Assessment (Ovid), SPORTDiscus (EBSCO), Scopus, Compendex (Engineering Village), INSPEC (Engineering Village), IEEE Xplore, and ACM Digital Library. Dissertations and Theses Global (ProQuest) were searched to identify dissertations or theses. The study registries searched were ClinicalTrials.gov and World Health Organization International Clinical Trials Registry Platform.

Search strategies included the use of text words and subject headings (eg, Medical Subject Headings and Emtree) related to five concepts: (1) stroke, (2) rehabilitation, (3) UE, (4) compensation, and (5) robotics or technology. The search was limited to English. Cochrane search filters were applied to exclude animal-only studies when possible [13]. All databases and registers were searched from the inception of resources. Searches were conducted on May 26, 2020. Searches were updated by rerunning all search strategies on July 23, 2021, and exporting new results. The full search strategies for each database and registry are provided in Multimedia Appendix 2.

### Study Selection

All search results were first imported into EndNote software, where duplicates were removed. The remaining results were imported into Covidence. A total of 2 screening steps were conducted: title and abstract screening and full-text screening. In all, 2 researchers (XW and YF) independently conducted title and abstract screening as well as full-text screening using the same inclusion and exclusion criteria. Disagreements between the 2 researchers were discussed and resolved between the 2 researchers. A third researcher (BY) was involved when an agreement could not be reached.

The inclusion and exclusion criteria used for the screening process are presented in Textbox 1.

**The inclusion and exclusion criteria used for the screening process.**

**Inclusion criteria**

- Stroke survivors or healthy participants enrolled in the intervention.
- The study involves upper extremity rehabilitation.
- Compensation was assessed using technology (ie, information and communication technologies, sensors, cameras, wearables, or artificial intelligence).
- The study involves compensation assessment or detection.
- The study involves compensation measurements: kinematic parameters (speed, angle, angular speed, etc), electromyogram, or compensatory posture or pattern classification.

**Exclusion criteria**

- Studies involving nonhuman participants.
- Studies about stroke neural recovery, stroke prevalence, and pathological analysis.
- Studies that do not use technology-based measurement methods.
- Studies on activity logs, functional electrical stimulation, gravity compensation in robotics, and effects of virtual therapy.
- Studies are not about upper extremity rehabilitation.
- Qualitative, usability, or nonacademic studies.
- Review studies such as systematic reviews.
- Case reports and letters.
- Studies are not written in English.

After the screening stage, studies were rated for their level of evidence based on the Centre for Evidence-Based Medicine (CEBM) [14] criteria. According to the CEBM, 4 clinical scales were used as reference standards, which included the compensation assessment scale—the Reaching Performance Scale [15], Motor Activity Log [16], Actual Amount of Use Test [17], and Chedoke-McMaster Stroke Assessment [18]. We used CEBM criterion 2b as a reference. The study would be regarded as having good reference standards if it had the same training task from any of the aforementioned 4 scales or if it had the same or partially the same evaluation criteria as any of the 4 scales.

## Results

### Overview

**Figure 1:**
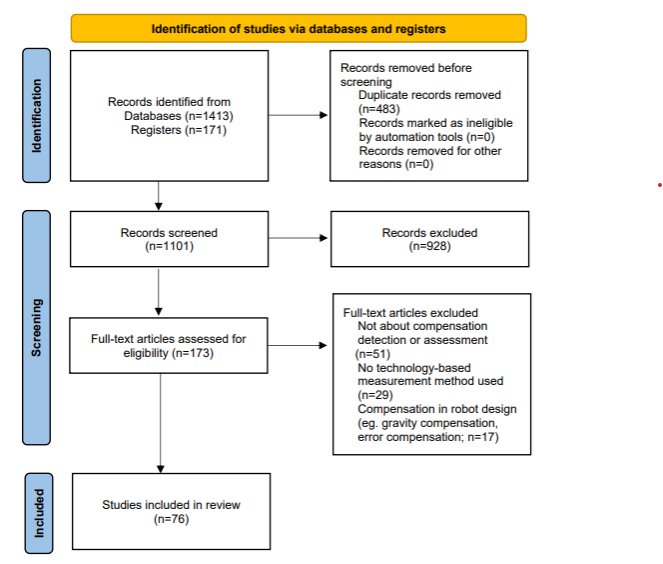
PRISMA (Preferred Reporting Items for Systematic Reviews and Meta-Analyses) flow diagram illustrating the screening process for papers included in this study.

A total of 1584 records were retrieved from the search. After removing duplicates, 69.51% (1101/1584) of records were screened at the title and abstract stage. In the first stage, 84.29% (928/1584) of the records were removed. The remaining 15.71% (173/1584) articles were subjected to full-text screening. A total of 76 studies were included after both screening stages. Figure 1 shows the PRISMA [19] flow diagram. After studies were rated based on the CEBM criteria, 72 (range from 1b to 2b in CEBM criteria) of the 76 (95%) studies were included in the final analysis; Table 1 shows the relationships between the included studies and the reference standards. In all, 67 papers were published after 2010, 69% (50/72) of which were published between 2015 and 2021.

**Table 1:** Correlation with reference standards.

Reference standard	Correlated references	Example
Reaching Performance Scale	[20-58]	[23]; task: reaching tasks; evaluation criteria: trunk displacement
Chedoke-McMaster Stroke Assessment and Reaching Performance Scale	[33,59-80]	[69]; task: a set of upper extremity exercises (Chedoke-McMaster Stroke Assessment); evaluation criteria: trunk displacement and shoulder movements (Reaching Performance Scale)
Motor Activity Log or Actual Amount of Use Test	[81-89]	[82]; task: activities of daily living; evaluation criteria: arm use

### Study Characteristics

Of the 72 studies, 38 (53%) recruited only stroke survivors, 9 (13%) included only healthy participants, and the remaining studies (n=25, 34%) recruited both (Table 2). Both men and women were included in most (48/72, 67%) studies. The age range of stroke survivors was 21 to 92 years and that of healthy participants was 18 to 85 years. For stroke survivors, the stage of recovery included subacute (between 1 and 6 months after stroke; 4 studies), chronic (>6 months after stroke; 36 studies), or both (18 studies). The sample size varied from 1 to 119 (Table 3).

**Table 2:** Characteristics of the studies (N=72).

		References
**Participants**		
	Stroke survivors	[21,23,25,26,28-30,32-34,38,40,44-47,51,52,54,56,58,60,62,65,67,69,71,73,74,76,77,79,80,82,85,86,90]
	Healthy participants	[20,31,39,42,43,55,59,61,78]
	Stroke survivors and healthy participants	[22,24,27,35-37,41,48-50,53,57,63,64,66,68,70,72,75,81,83,84,87-89]
**Stage of recovery**		
	Subacute	[35,73,82,89]
	Chronic	[21,23,26-30,32-34,38,40,41,44,46,47,51,52,56,58,60,62,65,68,69,71,74,75,79,80,83-85,87,88,90]
	Subacute and chronic	[22,24,25,36,37,45,50,53,54,57,63,64,66,67,72,77,86,91]

**Table 3:** The sample size distribution (N=72).

Sample size	Studies, n (%)
0-18	46 (64)
19-36	13 (18)
37-54	9 (13)
55-72	2 (3)
73-90	0 (0)
91-108	1 (1)
109-126	1 (1)

### RQ1: What Models Have Been Established to Assess and Detect Compensation?

#### Types of Compensation

##### Overview

Two types of compensation were identified according to the study by Miller et al [81]: (1) *disuse of the affected UE*, and (2) *use of the affected UE in an awkward manner to adjust for limited strength, mobility, and motor control.* We refer to the second type of compensation as awkward use of the affected UE for the remainder of this paper. Table 4 presents the compensation types, models, and measurements.

**Table 4:** Compensation type, model, and measurements.

Compensation type, model, and measurements					References
**Disuse of the affected upper limb**					
	**Arm use**				
		The ratio between the duration of movement in the least and less affected arm		[82]	
		Mean squared sum of the acceleration over a 1-minute epoch of the arm		[83,85]	
		Torques due to the measured tangential forces on the split-steering wheel		[84]	
	**Arm nonuse**				
		The difference of the Euclidean distance between the trunk and hand to the target		[86]	
		Movement time, peak velocity, total displacement, and movement smoothness		[87]	
		Root mean square of the rotation angle of the steering wheel		[88]	
		Total movement duration of each limb and the ratio between the movement duration in the paretic and nonparetic limb		[89]	
	Interlimb coordination	Amplitude, time, and frequency data from inertial sensors on upper body		[81]	
**Awkward use of the affected UE^a^**					
	**Trunk compensation**				
		**Trunk movements in the sagittal plane: trunk lean forward, trunk displacement, trunk flexion, trunk anteriorization, and trunk lean backward**			
			Trunk angular displacement	[20,22,23,25-27,39,47,60,63,68,90]	
			Trunk linear displacement	[24,30,40,41,51,52,58,66]	
			Trunk contribution slope	[37,38,62]	
			Acceleration of trunk motion	[28,64]	
			sEMG^b^ signal	[39,77]	
			Face orientation	[27]	
			Measurements for AI^c^-based compensatory posture classification	[43-45,48-50,53,54,61,74,77,91]	
			N/A^d^	[56,65,69]	
		**Trunk movements in the transverse plane: trunk rotation and trunk twist**			
			Trunk angular displacement	[22,25,26,39,47,68,90]	
			Acceleration of trunk motion	[27,28,64]	
			Trunk linear displacement	[34,40]	
			sEMG signal	[39]	
			Measurements for AI-based compensatory posture classification	[45,48-50,53,54,61,74,77,91]	
			N/A	[65,69]	
		**Trunk movements in the coronal plane: trunk leans from side to side, trunk contralateral and ipsilateral flexion, trunk lateral bending, and trunk lateral shift**			
			Trunk angular displacement	[21,22,47,60,68,90]	
			Trunk linear displacement	[34,41]	
			Measurements for AI-based compensatory posture classification	[61]	
		**Unspecified**			
			Trunk movement time, trunk distance, trunk peak velocity, and maximal angle of trunk flexion	[46]	
			Position and angle	[42,59]	
	**Shoulder compensation**				
		**Shoulder elevation**			
			Elevation angle of scapula, acromion, or acromio-clavicular joint	[26,39,57,66]	
			Acceleration of shoulder joint motion	[27,28]	
			Shoulder vertical translated distance	[37]	
			sEMG signal	[39]	
			Measurements for AI-based compensatory posture classification	[45,48-50,53,54,74,77,91]	
			N/A	[69]	
		**Shoulder abduction**			
			Acceleration of shoulder joint motion	[27,28,64]	
			Shoulder abduction angle	[70]	
			fMRI^e^	[71]	
		**Shoulder girdle compensatory movements**			
			Acceleration of shoulder joint motion	[29,64]	
			sEMG signal	[30]	
			Shoulder position	[31]	
			The coefficient of the elbow joint extension to the shoulder joint flexion ratio	[72]	
		Shoulder forward	Shoulder forward liner displacement	[26,32]	
		Shoulder overflexion	Shoulder flexion angle	[22,33,73]	
		Unspecified	Shoulder position	[59]	
	**Elbow compensation: insufficient elbow extension**				
		Elbow extension angle		[66,74,90]	
		Acceleration of elbow joint motion		[27,28]	
		N/A		[65]	
	**Finger compensation**				
		Individual finger compensation	Finger extension angle	[34]	
		**Multiple fingers adaptive compensation**			
			The covariance of individual finger impulses across multiple pulses	[75]	
			Pressure force of fingers	[76]	
	**Joint coordination**				
		Scapula, shoulder, elbow and wrist joint angles, movement time, goal-equivalent variance, nongoal-equivalent variance		[36]	
		Joint angles		[35]	
	Muscle synergy	sEMG signal		[33]	
	Slouching	Joint position		[69]	

^a^UE: upper extremity.

^b^sEMG: surface electromyogram.

^c^AI: artificial intelligence.

^d^N/A: not applicable.

^e^fMRI: functional magnetic resonance imaging.

##### Disuse of the Affected UE

A total of 9 studies assessed this type of compensation, and 3 models were discussed: the arm use model [82-85], arm nonuse model [86-89] and interlimb coordination model [81]. The arm use model measured the actual use of the impaired arm either in activities of daily living [82,83,85] or in bilateral and unilateral steering tasks [84]. The arm nonuse model was used to quantify the difference between the actual use of the impaired arm and its performance measured using standard clinical scales in reaching tasks [86,87], bilateral and unilateral steering tasks [88], and occupational therapy [89]. The interlimb coordination model was used to detect the reduction in interlimb coordination in stroke survivors compared with healthy participants in unimanual and bimanual activities of daily living [81]. In 4 studies [84,87-89], the tasks were completed using robot-assisted devices.

##### Awkward Use of the Affected UE

Most (63/72, 88%) studies assessed this type of compensation. The main models were (1) trunk compensation, (2) shoulder compensation, (3) elbow compensation, (4) finger compensation, and (5) others.

#### Trunk Compensation Model

This model (46/63, 73% studies) measures the awkward movements of the trunk for the affected UE [15]. Trunk compensatory movements can occur in 3 anatomical planes (sagittal, transverse, and coronal) of the human body. The sagittal plane (41/46, 89% studies) was the most common, which was described as trunk lean forward, trunk lean backward, trunk displacement, trunk flexion, and trunk anteriorization. The transverse plane (24/46, 52% studies) included trunk rotation and trunk twist. A total of 9 (20%) studies discussed the coronal plane, including trunk leans from side to side, trunk contralateral and ipsilateral flexion, trunk lateral bending, and trunk lateral shift (Table 3). The most common task (35/46, 76%) was the reaching task, followed by shoulder, elbow, and wrist exercises (4/46, 9%) [20,59-61], daily life activities [62-65], drinking tasks [66], simulated therapy activities [67], instrumented trunk impairment scale (version 2) tasks [21], Fugl–Meyer Assessment (FMA) items [22], occupational therapy tasks [68], and the Graded Repetitive Arm Supplementary Program (GRASP) [69], which is a set of UE exercises completed without the presence of a therapist. In 13 (28%) studies, tasks were completed using a robot-assisted device. In 4 (9%) studies, the tasks were conducted using VR [23-25] and mixed reality (MR) training systems [26].

#### Shoulder Compensation Model

This model (29/63, 46% studies) measures awkward movements of the shoulder of the affected UE [15], involving complex movements of the shoulder girdle and shoulder joint. The most observed shoulder compensation was shoulder elevation (17/29, 59% studies), followed by shoulder abduction [27,28,64,70,71], shoulder girdle compensatory movement [29-31,64,72], shoulder forward (protraction) [26,32], and shoulder overflexion [22,33,73]. The most commonly used task was reaching task (18/29, 62% studies). Other tasks involved hand-to-mouth tasks [33,70,73], drinking tasks [66,72], elbow ﬂexion-extension task [59,71], daily life activities [64,65], counterclockwise cyclic motions [31], FMA items [22], and GRASP [69]. In all, 12 studies were conducted using a robot-assisted device and 1 with an MR training system [26].

#### Elbow Compensation Model

This model measures awkward elbow movements of the affected UE [15]. A total of 6 studies found that stroke survivors had insufficient elbow extension during reaching tasks [27,28,74,90], drinking tasks [66], or daily life activities [65].

#### Finger Compensation Model

This model (3/63, 5% studies) measures the compensation among finger joints [34,75,76]. A study assessed the compensation among the joints in a finger in reaching tasks [34], whereas 2 other studies assessed compensation among multiple fingers in repetitive force-pulse tasks [75] and index finger movements [76].

#### Other Types of Compensation Models

Other types of compensation models included joint coordination [35,36], slouching [69], and muscle synergies [33], which were measured in reaching tasks, GRASP, and hand-to-mouth tasks, respectively.

### RQ2: What Measurements Are Used to Evaluate Compensatory Movements?

#### Disuse of the Affected UE

No standard measurement has been applied across studies for this type of compensation. For the arm use model, Ballester et al [83] and Hung et al [85] computed the mean squared sum of the acceleration over 1 minute. Thrane et al [82] calculated the arm movement ratio, that is, the ratio of arm use duration between the impaired arm and less impaired arm. Johnson et al [84] quantified the arm use by comparing the torque generated by the tangential forces of the 2 arms on the steering wheel.

For the arm nonuse model, Bakhti et al [86] computed proximal arm nonuse, which was the difference between the Euclidean distance between the trunk and hand to the target during the reaching movement in both spontaneous and maximal proximal arm use conditions. Johnson et al [87] used 4 kinematic metrics, including movement time, peak velocity, total displacement, and movement smoothness, to predict learned nonuse (LNU). Johnson et al [88] compared the root mean square of the rotation angle of the wheel in steering tasks in 3 different steering modes (unilateral nondominant, unilateral dominant, and bilateral) to quantify LNU. Barth et al [89] computed the total movement duration of each limb and the activity ratio, which was the movement duration of the paretic limb to the nonparetic limb to assess LNU.

Miller et al [81] created an array of numerical values, including amplitude, time, and frequency data from acceleration signals on the sternum, right wrist, left wrist, right upper limb, and left upper limb to characterize the interlimb coordination model.

#### Awkward Use of the Affected UE

The parameters for measuring trunk compensation in the sagittal plane included trunk angular displacement (12/41, 29% studies), trunk linear displacement (8/41, 20% studies), trunk contribution slope [37,38,62], acceleration of trunk motion [28,64], surface electromyogram (sEMG) signals [39,77], and face orientation [67]. The parameters used to measure trunk compensation in the transverse plane included trunk angular displacement (7/24, 29% studies), acceleration of trunk motion [27,28,64], trunk linear displacement [34,40], and sEMG signal [39]. A total of 2 parameters, trunk angular displacement (6/9, 67% studies) and trunk linear displacement [34,41] were measured to assess trunk compensation in the coronal plane.

The shoulder elevation compensation measurements included the elevation angle of the scapula, acromion, or acromioclavicular joint (4/17, 24% studies); acceleration of shoulder joint motion [27,28]; shoulder vertical translated distance [37]; and sEMG signal [39]. Shoulder abduction compensation was assessed by acceleration of shoulder joint motion [27,28,64], shoulder abduction angle [70], and functional magnetic resonance imaging (fMRI) [71]. Shoulder girdle compensatory movement measurements included acceleration of shoulder joint motion [29,64], sEMG signals [30], shoulder position [31], and the coefficient of the elbow joint extension to the shoulder joint flexion ratio [72]. A total of 3 studies [22,33,73] used the shoulder flexion angle to assess the shoulder overflexion compensation. The parameter for measuring shoulder forward compensation was shoulder forward liner displacement [26,32].

The elbow extension angle [66,74,90] and acceleration of elbow joint motion [27,28] were used to measure elbow compensation.

In all, 3 kinds of measurements were used to assess finger compensation. Fluet et al [34] measured the finger extension angle to assess the individual finger compensation. Kim et al [75] measured the covariance of individual finger impulses across multiple pulses, and Furudate et al [76] measured the pressure force of fingers to assess the compensation among multiple fingers.

As for other compensation models, Reisman and Scholz [36] measured multiple parameters including joint angles (ie, scapula, shoulder, elbow, and wrist), movement time, goal-equivalent variance, and nongoal-equivalent variance to evaluate joint coordination; Nibras et al [35] measured only joint angles to assess joint coordination. Lin et al [69] captured joint positions to assess slouching. Belfatto et al [33] measured sEMG signals to assess the muscle synergy.

### RQ3: What Types of Sensor Technology Are Used for Compensation Assessment and Detection?

#### Overview

A total of 6 types of sensors were identified as shown in Tables 5 and 6.

**Table 5:** Studies classified by sensor type (N=72).

Sensor type	Studies, n (%)
Body-worn sensor technology	25 (35)
Marker-based motion capture system	24 (33)
Marker-free vision sensor	16 (22)
Physiological signal sensing technology	10 (14)
Sensors embedded in rehabilitation training system	8 (11)
Ambient sensor	5 (7)

**Table 6:** Studies classified by sensor type (N=72).

Sensor type		Sensor measurement	Application settings			References
			Technology-based therapy setting	Home setting		
**Body-worn sensor**						
	Accelerometer	Acceleration of upper limb segments and trunk	[89]	[60,64,65,83]	[27,28,60,64,65,82,83,85,89]	
	IMU^a^	Original IMU signals or Euler angles of upper limb segments and trunk	[25,31,42,59,70]	[25,43,44,81]	[21,22,25,31,42-44,59,68,70,73,81,90]	
	Strain sensors	Electrical resistance of sensors printed on the stretched parts	N/A^b^	[78,79]	[78,79]	
	CyberGlove	Finger angles	N/A	N/A	[34]	
**Marker-based motion capture system**						
	Optical motion capture system	3D coordinates of the markers placed on the upper body	[24,26,31-33,38,39,42,45-47,59,62,63,87]	N/A	[24,26,31-33,36,38-40,42,43,45-47,57-59,62,63,66,72,87]	
	Electromagnetic motion capture system	3D coordinates of the markers placed on the upper body	N/A	N/A	[34]	
	Ultrasound 3D motion capture system	3D coordinates of the markers placed on the upper body	N/A	N/A	[86]	
**Marker-free vision sensor**						
	Microsoft Kinect depth sensor	Upper body joint positions in 3D space (x-y-z) coordinates	[23,41,48-52]	N/A	[20,23,41,48-52,61,69,86]	
	Simple camera	Video	[74,84]	[67]	[27,28,67,69,74,84]	
**Physiological signal sensing technology**						
	EMG^c^	sEMG^d^ signals of upper limb and trunk muscles	[29,30,33,39,54,84]	N/A	[29,30,33,39,53,54,77,84]	
	EEG^e^	EEG signals	[33]	N/A	[33,80]	
	fMRI^f^	fMRI images	N/A	N/A	[72]	
Sensors embedded in the training system	Force sensor or piezoelectric sensor or others	Force exerted by upper limbs, finger force, upper limb joint position, or orientation	[31,35,42,59,75,76,84,88]	N/A	[31,35,42,59,75,76,84,88]	
**Ambient sensor**						
	Pressure distribution mattress	Force distribution	[45,54,91]	[45,54,91]	[45,54,91]	
	Position sensor	Upper limb and trunk position	N/A	[55]	[55,56]	

^a^IMU: inertial measurement unit.

^b^N/A: not applicable.

^c^EMG: electromyogram.

^d^sEMG: surface electromyogram.

^e^EEG: electroencephalogram.

^f^fMRI: functional magnetic resonance imaging.

#### Body-Worn Sensor Technology

Body-worn sensors (25/72, 35% studies) were the most commonly used technology to detect compensatory movements, including accelerometers, inertial measurement units (IMUs), strain sensors, and CyberGlove. In all, 9 studies used accelerometers, including uniaxial [82] and triaxial [27,28,60,64,65,83,85,89]. Accelerometers were attached to different parts of the body. Some were worn on the wrists of both arms [82,83,89] or only on the wrist of the affected arm [85] to measure arm movement quantity, such as movement duration and acceleration magnitude, to evaluate arm use. Some were placed on the trunk (chest, middle back, or T12 vertebrae) [27,28,60,64], shoulder [27,28,64], elbow [27,28], and wrist [65] to measure time and movement variables, such as accelerations and joint angles of UE movement to detect trunk, shoulder, and elbow compensation. Among these studies, Antonio et al [27] and Carla et al [28] compared quantitative detection results using accelerometers with therapist-based visual analysis of video records. The results showed that the compensatory movements detected by the accelerometers, including shoulder abduction and elevation, insufficient elbow extension, and trunk forward displacement and rotation, were consistent with the therapists’ observations.

In all, 13 used IMUs. Each IMU typically consists of 1 or 2 triaxial accelerometers, a triaxial gyroscope, and a triaxial magnetometer [21,22,25,31,42,59,68,70,73,81]. The magnetometer was not included in some cases [43,44,90]. Accordingly, each IMU yielded 3D measurements of acceleration, angular velocity, and magnetic field vector (when using a magnetometer) in its intrinsic coordinate system [59]. In the reviewed studies, 1 to 9 IMUs were placed on the upper body parts, including the sternum [21,25,43,44,68,81,90], spine [21,22], pelvis [22], scapula [70,90], upper arms [22,25,31,42-44,59,70,73,81,90], forearms [22,25,43,44,59,70,90], wrists [73,81], and hands [22,70,90]. The original IMU signals [43,44,81] representing the movements of these body segments or the orientation in the form of Euler angles [21,22,25,31,42,59,68,70,73,90], of these body segments were output for compensation monitoring. It has been reported that IMUs can be used to detect trunk [21,22,25,42-44,59,68,90], shoulder [22,31,59,70,73], and elbow [22] compensation, as well as the interlimb coordination [81]. Furthermore, Ranganathan et al [43] proved that using IMUs could effectively detect compensatory trunk movements (approximately 90% accuracy) when compared with using an 8-camera motion capture system (Motion Analysis Corporation) as ground truth.

Moreover, 2 studies used changes in the electrical resistance of strain sensors printed on a garment [78,79] to identify different compensatory postures during UE movements. A study used CyberGlove to assess finger compensation by measuring the angles of finger joints [34]. Overall, 4 studies were conducted using robot-assisted therapies [42,59,70,87], 1 [25] using VR therapy, and 10 were conducted in homes [25,43,44,60,64,65,78,79,81,83].

#### Marker-Based Motion Capture System

The second most commonly used technology was the marker-based motion capture system (24/72, 33% studies). A total of 3 types of marker-based motion capture systems were used: an optical motion capture system (22/24, 92% studies), electromagnetic motion capture system [34], and ultrasound 3D motion capture system [86]. For this sensor, markers were attached to the upper body landmarks, which traditionally included the sternum, spinal process (C7 and T4), acromion processes, middle part of the humeri, lateral epicondyle, styloid process of the ulna, and bilateral thumbnails [62,63,87]. The participants were asked to perform the tasks while the positions of the markers were captured. The position and orientation of the trunk, shoulder, and elbow were then calculated according to the joint coordinate system method and used to characterize different compensation models.

For a long time, marker-based motion capture systems have been used as gold standard motion capture devices for clinical motion analysis [86]. Similarly, in the reviewed studies, marker-based motion capture systems were proven to be able to effectively identify compensation. In all, 5 studies have been used as the ground truth for the measurement of the effectiveness of other sensors in compensation detection [42,43,45,86,87]. Several interesting findings were reported using marker-based motion capture systems: (1) pre- and posttests showed that both robotic [32,38,63] and MR therapies [26] elicited benefits on reducing trunk compensatory movements, and stroke survivors showed less trunk compensatory movements during VR reaching [24]. However, Belfatto et al [33] argued that robotic therapy promoted the adoption of compensatory movements when stroke survivors performed training tasks; (2) therapist-based therapy [62] or a combination of robotic therapy and constraint-induced therapy [46] demonstrated more significant improvements in reducing trunk compensatory movements compared with robot-assisted therapy; (3) trunk displacement and shoulder elevation compensatory movements could discriminate between mild and moderate stroke paresis [66], whereas shoulder girdle compensatory movement could differentiate between mild or moderate and severe or pronounced stroke impairments [72].

Among all the studies, 13 [31-33,38,39,42,45-47,59,62,63,87] monitored compensation with robot-assisted upper limb devices, one [24] was conducted in VR therapy and one [26] was in MR therapy.

#### Marker-Free Vision Sensor

A total of 16 studies used marker-free vision sensors, including Microsoft Kinect depth sensors (versions 1 and 2) and a simple simple camera, as motion capture tools. Most (11/16, 69%) of these studies used Kinect, which is usually placed approximately 2.0 meters in front of the user to capture the 3D space (x-y-z) coordinates of 20 (version 1) or 25 (version 2) skeleton joint positions in the user’s body at 30 frames per second. In the reviewed studies, the locations and orientations of the upper body parts (ie, hip, spine, shoulder, elbow, wrist, and hand) [48-50,61,69,86] or spine [20,23,41,51,52] were recorded, and 2 studies have verified the effectiveness of this sensor technology for monitoring compensation. Bakhti et al [86] proved that Kinect can be used to accurately assess proximal arm nonuse when compared with an ultrasound 3D motion capture system (CMS20s, Zebris). The agreement between Kinect and CMS20s was measured using intraclass correlation coefficients (0.96), linear regression (*r*^2^=0.92), and Bland and Altman plots (Kinect: −4.25, +6.76 to –6.76); CMS20s: −4.71, +7.88 to –7.88). Lin et al [69] found substantial agreement of detected compensation, such as shoulder elevation and hip extension, between annotated videos and Kinect (Cohen *κ* 0.60-0.80) and almost perfect agreement for trunk rotation and flexion (Cohen *κ* 0.80-1).

Overall, 6 studies used RGB cameras and 2 (33%) of them [67,74] used a camera to collect motion images to extract kinematic data through third-party data extraction algorithms for quantitative compensation assessment. The other 4 (67%) studies collected motion videos for clinicians’ visual evaluation of compensation.

In all, 8 studies [41,48-52,74,84] were conducted using robot-assisted upper limb devices, 2 studies [23,69] were conducted using VR therapy, and 1 study [67] was conducted in a home using a single low-cost camera.

#### Physiological Signal Sensing Technology

Physiological signal sensing technologies include electromyogram (8/72, 11% studies), electroencephalogram (EEG) [33,80], and fMRI [71] systems. According to the reviewed studies, sEMG signals of upper limb muscles (including, but not limited to, biceps, triceps, upper trapezius, pectoralis major, brachioradialis, anterior, middle, and posterior deltoids) and trunk muscles (left or right rectus abdominis, left or right obliquus externus abdominis, left or right thoracic erector spinae, left or right lumbar erector spinae, and descending part of the trapezius) not only helped to discriminate true recovery and compensation [29,30,33,84] but also could be used as features for automatic compensation detection [39,53,77]. Chen et al [77] confirmed that using a generative adversarial network with sEMG signals as features could achieve excellent detection performance (accuracy=94.58%, +1.15% to –1.15%) of trunk compensatory movements.

A study used fMRI [71] to study the cortical activation pattern of compensatory movements and demonstrated that compensatory movements require a greater recruitment of cortical neurons. A total of 2 studies [33,80] showed that brain scalp EEG signals could help researchers gain more insight into the relationship between motor compensation and underlying brain activities. Among all studies, electromyogram [29,30,33,39,54,84] and EEG [33] systems were used along with robot-assisted devices for compensation assessment.

#### Sensors Embedded in the Rehabilitation Training System

In all, 8 studies directly selected sensors embedded in the rehabilitation training system as compensation evaluation tools. Nibras et al [35] used the measurement information in an exoskeleton to distinguish between recovery and compensation in stroke survivors. They found 2 compensatory patterns in stroke survivors: atypical decoupling of the shoulder elevation and forearm joints and atypical coupling of the shoulder horizontal rotation and elbow joints, by analyzing 4 ArmeoSpring angles when stroke survivors performed reaching movements with the ArmeoSpring exoskeleton. In contrast, a simpler and less complex UE rehabilitation robot, such as an end-effector robot, may not have the capacity to provide detailed UE measurement information as the exoskeleton. Therefore, additional sensors, such as inertial sensors [31,42,59], are required with the sensors in the end-effector robot to satisfy compensation assessment needs. In addition, Johnson et al [84,88] used the force sensors of a UE rehabilitation system, a driver simulation system, to quantify impaired arm activity [84] and LNU [88]. Kim et al [75] and Furudate et al [76] used force sensors in hand rehabilitation systems to evaluate the compensation among individual fingers.

#### Ambient Sensors

A total of 5 studies used ambient sensors, including a pressure distribution mattress (Body Pressure Measurement System, Model 5330, Tekscan, Inc) [45,54,91] and position measurement sensors [55,56]. A pressure distribution mattress was used to measure a person’s body pressure distribution in a seated position for the automatic detection of compensatory postures [45,54,91]. Cai et al [45] verified the effectiveness of using pressure distribution data together with machine learning (ML) algorithms to detect compensatory patterns using a 3D motion capture system (VICON, Oxford Metrics) as the ground truth. When using a pressure mattress or VICON, the average *F*_1_ score (an evaluator of the ML algorithm performance) was >0.95.

The position measurement sensors used were either a force sensor placed anterior to the back of the chair [56] or a contactless first-reflection ultrasonic echolocation sensor placed on the edge of a table [55] to monitor the position of the trunk. As only the trunk position was monitored, the researchers only realized a rough detection of compensatory trunk flexion movement. In addition, 3 studies [45,54,91] used robot-assisted upper limb devices, and 4 studies have proposed that these systems could be used in a home environment [45,54,55,91].

### RQ4: Which Statistical or AI Methods Have Been Used for Compensation Assessment and Detection?

#### Overview

Overall, 56 studies used statistical methods and 15 adopted AI-based methods as shown in Table 7 and Table 8, respectively.

**Table 7:** Studies classified by statistical methods (N=56).

Data analysis scenario and statistical method			References
**Differences among groups**			
	ANOVA	[24,36-38,41,46,75,84,88]	
	Mean and SD	[20,24,27,37,40,89]	
	Mann-Whitney *U* test	[36,40,66,85]	
	Wilcoxon test	[40,66]	
	Paired-sample *t* test	[88], 1-tailed; [66], 2-tailed; [73], 2-tailed	
	Principal components analysis	[35,36,66]	
	Regression analysis	[40,73,76]	
	Tukey honestly significant difference post hoc analysis	[24,37]	
	Tukey-Kramer tests	[84]	
	Scheffé test	[75]	
	Log-modulus transformation methods	[75]	
	Nonparametric Friedman test	[40]	
	Independent-samples *t* test	[66]	
	Kolmogorov-Smirnov normality test	[37]	
	Spearman rank correlations	[90]	
	Pearson correlations	[85]	
	Bonferroni corrections	[85]	
	Chi-square test	[85]	
	Graph learning theory	[22]	
**Differences before and after the intervention**			
	Wilcoxon signed-rank test	[32,33,52,72]	
	Mean and SD	[58,62,63]	
	ANOVA	[29,34,56]	
	Spearman rank correlation coefficient	[63,72]	
	Tukey HSD^a^ test	[29,56]	
	Analysis of covariance	[52,62]	
	2-sample and paired *t* tests	[52], 1-tailed; [58], 2-tailed	
	Pearson correlation coefﬁcient	[33]	
	Kolmogorov-Smirnov test	[58]	
	Mann-Whitney *U* test	[72]	
**Real time changes**			
	Canonical correlation analysis	[26,70,80]	
	Mean and SD	[21,23,28,31,42,47,51,57,59,60,64,68,69,82,83,86]	
	ANOVA	[25]	
	Spearman correlation test	[65,81,82,86,87]	
	Logistic regression	[65,82]	
	Paired *t* test	[87], 2-tailed	
**Associations of physiological signals with compensation parameters**			
	Spearman rank correlation coefﬁcient test	[71]	
	Pearson correlation test	[39]	
	ANOVA	[30]	
	Post hoc contrasts	[30]	

^a^HSD: honestly significant difference.

**Table 8:** Studies classified by machine learning (ML) algorithms (N=15).

ML algorithm and accuracy		References
**Linear SVM^a^**		
	Health: trunk compensation in 3 directions (AUC)^b^=99.15%	[61]
	Stroke (*F*_1_ score): NC^c^=0.88; SE^d^=0.86; TR^e^=0.80; LF^f^=0.81	[77]
	Healthy group (AUC): NC=0.86; SE=0.68; TR=0.74; LF=0.98 and stroke group (AUC): NC=0.63; SE=0.27; TR=0.82; LF=0.92	[48]
	Healthy participant (*F*_1_ score): NC=0.87; SE=0.15; TR=0.5; LF=0.74 and stroke survivor (*F*_1_ score): NC=0.94; SE=0; TR=0; LF=0	[49]
	Healthy group (AUC): NC=0.98; SE=1.00; TR=0.99; LF=0.97 and stroke group (AUC): NC=1.00; SE=0.98; TR=0.85; LF=0.90	[53]
	Stroke (*F*_1_ score): NC=0.990; SE=0.975; TR=0.983; LF=0.975	[54]
	Stroke: offline (*F*_1_ score): NC=0.984; SE=1.000; TR=0.995; LF=0.963 and on the web: participant 1 (*F*_1_ score): NC=0.978; SE=1.000; TR=0.929; LF=1.000; participant 2 (*F*_1_ score): NC=0.994; SE=1.000; TR=1.000; LF=0.984	[45,91]
	Stroke: trunk flexion (AUC)=78.2%	[55]
***k*-NN^g^**		
	Health: trunk compensation in 3 directions (AUC)=97.9%	[61]
	Stroke (*F*_1_ score): NC=0.79; SE=0.78; TR=0.70; LF=0.73	[77]
	Health: correct vs incorrect (involving typical compensatory movements) upper limb exercises (sensitivity and specificity): garment 1: 86%, +6% to –6% vs 79%, +7% to –7%; garment 2: 89%, +6% to –6% vs 93%, +5% to –5%; garment 3: 87%, +4% to –4% vs 84%, +4% to –4%	[78]
	Health: 3 incorrect compensatory positions (not specified) in UE^h^ adduction exercise (*k* value): pos_run1=0.78, pos_run2=0.82, pos_run3=0.79, pos_run4=0.81	[79]
	Stroke (*F*_1_ score): NC=0.989; SE=0.970; TR=0.983; LF=0.981	[54]
**Naïve Bayes**		
	Health: trunk displacement (precision and Recall)—non-compensatory=92.7% and 90.5% and compensatory=88.6% and 91.2%	[43]
	Stroke: trunk compensatory movements in anterior and posterior direction (precision)—Horizontal Reach: unaffected arm=100%, affected arm=87.5%; Vertical Reach: unaffected arm=87.5%, affected arm=100%; Card Flip: unaffected arm=62.5%, affected arm=66.7%; Jar Open: unaffected arm=71.4%, affected arm=71.4%	[44]
**Logistic regression**		
	Healthy: trunk compensation in 3 directions (AUC)=83%	[61]
	Health: 3 incorrect compensatory positions (not specified) in UE adduction exercise (*k* value): pos_run1=0.82, pos_run2=0.85, pos_run3=0.88, pos_run4=0.89	[79]
Random Forest	Healthy: trunk compensation in 3 directions (AUC)=96%	[61]
Multilabel *k*-NN	Stroke (*F*_1_ score): NC=0.73; SE=0.53; TR=0.67; LF=0.69; insufficient elbow extension=0.73	[74]
Multilabel decision tree	Stroke (*F*_1_ score): NC=0.69; SE=0.50; TR=0.60; LF=0.68; insufficient elbow extension=0.80	[74]
Generative adversarial network *k*-NN	Stroke (*F*_1_ score): NC=0.94; SE=0.95; TR=0.93; LF=0.96	[77]
Sequential minimal optimization	Stroke: trunk compensatory movements in anterior and posterior direction (precision)—horizontal reach: unaffected arm=85.7%, affected arm=87.5%; vertical reach: unaffected arm=100%, affected arm=100%; Card Flip: unaffected arm=62.5%, affected arm=66.7%; Jar Open: unaffected arm=57.1%, affected arm=57.1%	[44]
Decision tree J48	Health: 3 incorrect compensatory positions (not specified) in UE adduction exercise (*k* value): pos_run1=0.64, pos_run2=0.81, pos_run3=0.82, pos_run4=0.81	[79]
Recurrent Neural Network	Healthy group (AUC): NC=0.87; SE=0.79; TR=0.84; LF=0.98 and stroke group (AUC): NC=0.66; SE=0.27; TR=0.81; LF=0.77	[48]
Weighted random Forest	Healthy participant (*F*_1_ score): NC=0.87; SE=0.15; TR=0.5; LF=0.74 and stroke survivor (*F*_1_ score): NC=0.94; SE=0; TR=0; LF=0	[49]
Cost sensitive	Healthy participant (*F*_1_ score): NC=0.83; SE=0.09; TR=0.19; LF=0.68 and stroke survivor (*F*_1_ score): NC=0.94; SE=0; TR=0; LF=0	[49]
Random Undersampling	Healthy participant (*F*_1_ score): NC=0.71; SE=0.29; TR=0.48; LF=0.72 and stroke survivor (*F*_1_ score): NC=0.69; SE=0.04; TR=0.20; LF=0.07	[49]
Tomek links	Healthy participant (*F*_1_ score): NC=0.79; SE=0; TR=0; LF=0 and stroke survivor (*F*_1_ score): NC=0.94; SE=0; TR=0; LF=0	[49]
SMOTE^i^	Healthy participant (*F*_1_ score): NC=0.72; SE=0.3; TR=0.49; LF=0.82 and stroke survivor (*F*_1_ score): NC=0.83; SE=0.06; TR=0.25; LF=0.01	[49]
SVM SMOTE	Healthy participant (*F*_1_ score): NC=0.66; SE=0.28; TR=0.49; LF=0.73 and stroke survivor (*F*_1_ score): NC=0.8; SE=0.04; TR=0.24; LF=0.05	[49]
Random oversampling	Healthy participant (*F*_1_ score): NC=0.77; SE=0.32; TR=0.51; LF=0.63 and stroke survivor (*F*_1_ score): NC=0.8; SE=0.04; TR=0.23; LF=0.07	[49]
Binary classification	Healthy participant (AUC)—good example: SE=0.94; TR=0.97; LF=0.92; bad example: SE=0.37; TR=0.63; LF=0.52	[50]

^a^SVM: support vector machine.

^b^AUC: area under the curve.

^c^NC: no compensation.

^d^SE: shoulder elevation.

^e^TR: trunk rotation.

^f^LF: lean forward.

^g^*k*-NN: *k*-nearest neighbor.

^h^UE: upper extremity.

^i^SMOTE: synthetic minority oversampling technique.

#### Statistical Methods

Statistical methods were used to assess compensation from 4 perspectives: real time changes of compensation measurements in body movements, group variance in compensation measurements, effects of an intervention on compensation measurements, and the statistically significant associations of physiological signals with compensation measurements.

A total of 23 studies used mean and SD, canonical correlation analysis, Spearman correlation, step-wise multiple regression, or ANOVA to test the real time changes of compensation measurements in body movements. For instance, Wittmann et al [25] used repeated measures 1-way ANOVAs to test trunk orientation changes during rehabilitation training to assess trunk compensation in real time.

In all, 20 studies tested the differences among groups to assess compensation. The most commonly used statistical methods were ANOVA and Mann–Whitney *U* test. For instance, Kim et al [75] compared all compensation measurements between groups (stroke survivors vs healthy participants) and between hands (within-group factor: more affected hand vs less affected hand in stroke survivors and nondominant hand vs dominant hand in healthy participants) with ANOVA for compensation assessment.

In addition, 10 studies analyzed the differences in compensation measurements before and after the intervention. Wilcoxon signed-rank test, ANOVA, Spearman rank correlation coefficient, paired 1- and 2-tailed *t* test and 1- and 2-tailed Tukey honestly significant difference tests were used in these studies. For instance, Fluet et al [34] used ANOVA to analyze how 2 different training models (traditional vs VR-based training) affect upper limb compensation in the dimensions of peak reaching velocity, finger extension excursion, shoulder excursion, elbow excursion, and trunk excursion.

Overall, 3 studies tested the associations between physiological signals, such as fMRI and sEMG, and compensation parameters [30,39,71]. For instance, Lee et al [71] used Spearman rank correlation coefﬁcient to test the relationship between the brain activation area and shoulder abduction angle. They found that greater activation of the supplementary motor area was required for a larger shoulder abduction angle. Huang et al [39] applied the Pearson correlation test and found a positive correlation between muscle fatigue (measured by sEMG median frequency) and compensation. They concluded that sEMG median frequency was a good indicator of compensation due to muscle fatigue.

#### AI-Based Methods

A total of 15 studies used AI-based methods to detect compensatory postures, and 9 studies classified 3 common compensatory postures: trunk lean forward, trunk rotation, and shoulder elevation [45,48-50,53,54,74,77,91]. In addition, 4 studies discriminated trunk compensatory movements in the sagittal, transverse, and coronal planes [43,44,55,61], and 2 studies did not mention the type of compensatory posture that was classified [78,79]. Dolatabadi et al [50] made the compensation data set public for other researchers. A total of 2 studies used this data set to train their ML models to improve the accuracy of compensation detection [48,49]. The remaining studies collected their own data to detect compensation.

Various ML algorithms were applied to train the classification model (Table 6). The most commonly used ML algorithm was the support vector machine (SVM). Cai et al [45] reported the highest average *F*_1_ score (0.99) for recognizing trunk lean forward, trunk rotation, and shoulder elevation based on 5 features extracted from the pressure distribution data. Nordin et al [61] reported the highest accuracy (99.15%) for detecting the 3D trunk compensatory postures.

Notably, 8 studies [44,48,49,54,61,74,77,79] used more than one ML algorithm to compare the classification results for compensatory postures. For example, Zhi et al [48] used both SVM and recurrent neural network classifiers to classify shoulder elevation, trunk rotation, and lean forward. The results demonstrated high accuracy in healthy participants, but low accuracy in stroke survivors. Cai et al [54] applied the *k*-nearest neighbor and SVM algorithms to detect and categorize shoulder elevation, trunk rotation, and lean forward in stroke survivors, and both algorithms yielded high classification accuracies (*F*_1_ score >0.95). Nordin et al [61] used 4 different classiﬁcation algorithms with 10-fold cross-validation to assess the 3D trunk compensatory movements. The results showed accuracy of 99%, 98%, 96%, and 83% with SVM, *k*-nearest neighbor, random forest, and logistic regression, respectively.

## Discussion

To the best of our knowledge, this is the first systematic review of technologies for compensation assessment and detection of UE movements in stroke survivors.

### RQ1: What Models Have Been Established to Assess and Detect Compensation?

Notably, 2 types of compensation were categorized. Most (63/72, 88%) studies focused on investigating the awkward use of the affected UE. The reason might be that the awkward pattern is more complicated to be observed than the disuse pattern [81]. The synergy and coupling of body parts are difficult to understand [92], which requires more evidence-based methods to fuse data from more resources across a constant timeline. Sensor technologies offer fine-grained rich data, and together with AI methods, can provide a low-cost solution for continuous monitoring of a person’s performance.

The models of the disuse pattern focus on the amount of use of the affected UE. For the awkward pattern, the models focused more on how the unaffected body parts were involved in the motion with the affected UE. The most discussed body parts were the trunk, shoulders, and elbows. Trunk compensation was the most discussed factor, suggesting that it is more common among stroke survivors.

Models were established for different task scenarios. For the disuse pattern, bilateral tasks were the most common. For the awkward pattern, reaching tasks were mostly used. Reaching was the basic movement of the upper limbs that constituted most daily life behaviors [93]. Reaching requires coordination of multiple joints of the arm and is controlled by the central nervous system [93]. Different reaching ranges can result in various compensations for the trunk, shoulder, and elbow [15].

### RQ2: What Measurements Are Used to Evaluate Compensatory Movements?

Notably, 2 clinical scales, the Motor Activity Log [16] and the Actual Amount of Use Test [17], have traditionally been used for the evaluation of disuse patterns. However, these are subjective and difficult to replace using technology-based methods. Levin et al [15] proposed the Reaching Performance Scale for awkward pattern evaluation. However, none of the reviewed studies have quantified this scale using technological methods. Moreover, UE functional impairment scales (eg, FMA) were not used to assess compensation.

Quantitative measurements have been proposed for technology-based compensation assessments. For the disuse pattern, measurements such as the movement duration and frequency of use were used to describe the use of the affected UE. For the awkward pattern, linear displacement, angular displacement, acceleration, and sEMG signals of the trunk and upper limb joints were the most common measurements. Furthermore, the trunk compensation measurements, which are the kinematic measurements of the trunk in the 3 anatomical planes, are more uniform. In contrast, shoulder compensation measurements are more diverse and complex. This could be because the shoulder has more freedom of movement, and the configuration of these movements could vary across different experimental tasks [26-28,37,39,45,48-50,53,54,64,66,69,91].

Further studies could be conducted to explore the relations among all these compensation measurements and to develop a set of gold standard quantitative measurements.

### RQ3: What Types of Sensor Technology Are Used for Compensation Assessment and Detection?

Marker-based motion capture systems yield accurate and robust real time motion tracking and have been used as ground truth to verify the effectiveness of other sensors for compensation assessment and detection [42,43,45]. In our reviewed studies, marker-based motion capture systems were used to detect various compensations, including arm nonuse [86,87], trunk compensation [26,45], shoulder compensation [66,72] and interlimb coordination [36]. The drawbacks of these systems include but are not limited to the cost of both hardware and software, complicated setup, and the need for professionals to operate the systems [50]. These systems may also require a specific space, such as an area with a clear line of sight for the cameras [44]. The use of cameras in a home environment may raise privacy concerns [44].

Similarly, although with great accuracy, the setup of physiological signal sensing technologies is complex and has been limited to its use in laboratories or other controlled environments. In addition, professionals are required to collect and analyze these physiological signals [27]. The advantage of using this sensor technology is that the recorded sEMG signals of relevant muscles [30,84], brain scalp EEG signals [33,80], and cortical activation patterns [71] could help researchers gain more insight into compensation from the perspective of muscle activities and brain activities, which in turn would provide more information for compensation detection and correction to improve UE motor performance in stroke survivors.

Body-worn sensors were the most common technology used for compensation assessment and detection in the reviewed studies. They were able to monitor all compensation models [27,28,42-44,70,81-83]. Compared with marker-based motion capture systems, body-worn sensors are more affordable and portable, with a simpler setup [43,44]. More than half (47/72, 65%) of the studies used these sensors in technology-based therapies or home settings, which shows that this sensor technology has great potential for use in less-supervised therapy environments. The main disadvantage of this technology is that it can induce unnatural movements owing to the sensor attachment on the user’s body, which may affect the accuracy of compensation assessment [45]. Future research could focus on reducing or avoiding the possible unnatural movements caused by sensor attachment during a compensation assessment process, such as correcting the deviation through algorithms or adopting a more ingenious physical layout of the sensors.

Similar to body-worn sensors, marker-free vision sensors are low-cost and have an easy setup [94]. Owing to their size and portability, they could be an ideal option for home use. Marker-free vision sensors have been used to detect arm use [84]; arm nonuse [86]; and trunk [20,23], shoulder [69,74], and elbow [74] compensation. They were used together with ML algorithms to automatically detect typical compensatory postures (no compensation, shoulder elevation, trunk rotation,

lean forward, etc) [48,49,61,74]. The sensors can capture stroke survivors’ motion images in real time for clinicians to determine the compensation adopted during the training process. These images were used to train AI models to automatically detect compensatory postures. Compared with the RGB camera, Kinect was more commonly used. This could be because of the various types of information provided by the Kinect depth sensor, including color images, depth images, and 3D skeleton joint positions of the human body. However, it has been reported that the prediction of joint positions of the shoulder and trunk by Kinect suffers from large errors when sitting with trunk flexion (approximately 100 mm), which is a common compensatory movement after stroke [61]. One of the weaknesses of using marker-free vision sensors is that they can introduce privacy concerns if used in a home and may induce unnatural behaviors owing to the negative feelings caused by surveillance [44].

Relatively few studies have been conducted on sensors embedded in rehabilitation systems and ambient sensors for compensation assessment and detection. When a stroke survivor completes exercises with the assistance of a rehabilitation training system, it is intuitive to use the same system for compensation assessment [95]. However, for less complex rehabilitation robots with a simpler setup, such as end-effector robots, external measures may be required because the data collected by the system are not sufficient to detect compensation [31,42,59]. Ambient sensors are typically simple and unobtrusive [45]. They have great potential for use in compensation assessment and detection in less-supervised therapy environments, especially in home settings. However, only limited compensation can be detected by ambient sensors. Thus, more research could focus on accurately detecting compensatory movements using these sensors.

In summary, all sensor technologies have their own advantages and disadvantages. Both marker-based motion capture systems and physiological sensing technologies are limited by their use of a more controlled environment. Although with great accuracy in compensation detection, the setup is complicated and requires expert experience. Marker-based technology is usually used as the gold standard to test the accuracy of other technologies in compensation detection and measurement. In comparison with the results of marker-based technologies, body-worn sensors [27,28,43], marker-free vision sensors [86], sensors embedded in rehabilitation training systems [42], and ambient sensors [45] have also been proven effective in compensation assessment and detection. Body-worn sensors, marker-free vision sensors, and ambient sensors are low-cost, easy to set up, and can be used in less-controlled environments, such as home settings. However, marker-free vision sensors can increase privacy concerns. Thus, it may cause deployment issues in the home environment. Both wearable sensors and marker-free vision sensors can cause incorrect postures owing to the unnatural movements induced by the sensors. Directly using sensors embedded in rehabilitation training systems to assess and detect compensation could be a simple and convenient method. However, researchers should be aware of (1) whether the sensors in the system can meet the accuracy requirements and (2) whether the sensors in the system can capture all the necessary data for compensation assessment and detection. Finally, it is suggested that a rehabilitation training system be built that integrates training exercises, compensation assessment and detection, and real time compensation feedback for stroke survivors to perform effective rehabilitation with less or even without the supervision of a therapist.

### RQ4: Which Statistical or AI Methods Have Been Used for Compensation Assessment and Detection?

Research based on statistical methods provides valuable information about compensation assessment and detection, such as the difference in compensation measurements between healthy people and stroke survivors [20,66,73,84], changes in compensation measurements before and after an intervention [34,62,63,72], and the correlation of physiological signals with compensation measurements [30,39,71]. This information can be processed further in future studies for compensation assessment and detection.

The majority of studies used descriptive statistics, such as mean and SD, for real time compensation detection [23,47,68,69,82,83,86]. Although descriptive statistics are simple to use, the application of this method to detect compensation relies heavily on expert experience. For example, an acceptable range of compensation measurements was set by therapists, and the occurrence of compensation was decided by the therapists based on observation of the stroke survivors’ movements if they exceeded the compensation range. Therefore, this method is subjective and may not be accurate. In future, more research could focus on using other statistical methods, such as logistic regression, for real time detection of compensatory movements.

In contrast to statistical methods, AI methods have been used to automatically detect compensatory postures. They showed great potential for real time compensatory posture detection in less-supervised therapy environments [43-45,48-50,53,54,74,78,79,91]. One limitation of this research area is that there are few public data sets on compensatory movements in stroke survivors. In our review, only one open data set (the Toronto Rehab Stroke Posture data set) was found. Open research data are an originally collected data set that is accessible and can be reused by other researchers to conduct their research [96,97]. It has been gaining attention and growing popularity among researchers and funding agencies [96,98]. As such, future studies should make data accessible and sharable among research communities.

Furthermore, although a variety of ML algorithms have been identified for compensatory posture detection, they can only identify a single compensatory posture at a time, which cannot meet the situation where multiple compensatory postures appear concurrently. Moreover, AI methods have not yet been used to predict the occurrence of compensation. Therefore, more effort is needed to build more heterogeneous AI models, such as multilabel ML models and deep learning models, for multiple compensation detection and prediction.

### Strengths and Limitations

Our study had several strengths. This study applied comprehensive searches in both technology and medical fields. This is the first comprehensive systematic review of technology-based compensation assessment and detection in UE rehabilitation for stroke survivors. It is the only systematic review summarizing compensation models and their measurements and has reviewed the use of statistical and AI methods for compensation assessment and detection.

Our study has some limitations. First, the review included only references in English. Second, owing to inconsistencies in compensation assessment criteria across studies, the review did not include comparisons of the effectiveness of different technologies for compensation evaluation.

### Conclusions and Future Research

This systematic review focuses on how technologies are used for compensation assessment and detection during UE rehabilitation of stroke survivors. It covers models and measurements to describe the compensation and different types of sensors and statistical and AI methods for compensation assessment and detection. Evidence suggests that technology-based compensation assessment and detection can augment rehabilitation without the constant presence of therapists. Future studies could (1) explore how to develop a set of gold standard quantitative compensation measurements; (2) investigate how to overcome the discussed defects of body-worn sensors, marker-free vision sensors, and system-embedded sensors in compensation evaluation and how to integrate feedback with these sensors so that they can be used in less-supervised or even unsupervised UE rehabilitation environments; (3) focus more on open data as they provide opportunities for reuse in algorithm development for automatic real time compensation assessment and detection; (4) study multilabel classification algorithms and deep learning algorithms for multiple compensation detection; and (5) research more on compensation prediction.

## Appendix

Multimedia Appendix 1 PRISMA (Preferred Reporting Items for Systematic Reviews and Meta-Analyses) checklist.

Multimedia Appendix 2 Search strategy.

## Abbreviations

AI: artificial intelligence
CEBM: Centre for Evidence-Based Medicine
EEG: electroencephalogram
FMA: Fugl–Meyer Assessment
fMRI: functional magnetic resonance imaging
GRASP: Graded Repetitive Arm Supplementary Program
IMU: inertial measurement unit
LNU: learned nonuse
ML: machine learning
MR: mixed reality
PRISMA: Preferred Reporting Items for Systematic Reviews and Meta-Analyses
RQ: research question
sEMG: surface electromyogram
SVM: support vector machine
UE: upper extremity
VR: virtual reality
