# Supplementary material for: Technology-Based Compensation Assessment and Detection of Upper Extremity Activities of Stroke Survivors: Systematic Review
Source: J Med Internet Res. 2022 Jun 13;24(6):e34307. doi: 10.2196/34307 (PMC9237771; doi:10.2196/34307)
Supplement: Multimedia Appendix 2 [file jmir_v24i6e34307_app2.docx]

**Supplement A: Search Strategies**

Medline ALL (Ovid) Search Strategy

1 cerebrovascular disorders/ or exp basal ganglia cerebrovascular disease/ or exp brain ischemia/ or exp carotid artery diseases/ or exp intracranial arterial diseases/ or exp intracranial arteriovenous malformations/ or exp "intracranial embolism and thrombosis"/ or exp intracranial hemorrhages/ or vasospasm, intracranial/ or vertebral artery dissection/

2 exp Stroke/

3 (stroke? or poststroke? or post-stroke? or cerebrovasc* or brain vasc* or cerebral vasc* or cva? or apoplex* or SAH? or TIA?).tw,kf,jw.

4 Transient Ischemic Attack*.tw,kf.

5 ((brain* or cerebr* or cerebell* or intracran* or intracerebral) adj5 (isch?emi* or infarct* or thrombo* or emboli* or occlus*)).tw,kf.

6 ((brain* or cerebr* or cerebell* or intracerebral or intracranial or subarachnoid) adj5 (haemorrhage* or hemorrhage* or haematoma* or hematoma* or bleed*)).tw,kf.

7 hemiplegia/ or exp paresis/

8 (hemipleg* or hemipar* or paresis or paretic).tw,kf.

9 or/1-8

10 Stroke Rehabilitation/

11 Rehabilitation/

12 Rehabilitation Centers/

13 Neurological Rehabilitation/

14 exp Exercise Therapy/

15 telerehabilitation/

16 rh.fs.

17 (rehab* or neurorehab* or telerehab*).tw,kf,jw.

18 exp Physical Therapy Modalities/

19 (physical adj2 therap*).tw,kf.

20 physiotherap*.tw,kf.

21 physio-therap*.tw,kf.

22 Kinesiotherap*.tw,kf.

23 exp Exercise/

24 exp Exercise Movement Techniques/

25 (exercise* or exercising).tw,kf.

26 ((musculoskeletal or muscle? or cardio* or cardiac* or strength* or enduranc* or balanc* or gait* or task? or coordination) adj2 (train* or retrain* or therap* or intervention*)).tw,kf.

27 or/11-26

28 exp Upper Extremity/

29 (upper limb* or upper extremit* or upper body or arm* or shoulder* or hand* or axilla* or elbow* or forearm* or finger* or wrist*).tw,kf.

30 (dexterity or dexterous*).tw,kf.

31 (grip or gripping).tw,kf.

32 (grasp or grasping).tw,kf.

33 (tap or tapping).tw,kf.

34 (reach or reaching).tw,kf.

35 or/28-34

36 Robotics/

37 robot*.tw,kf,jw.

38 Automation/

39 automat*4.tw,kf.

40 (powered adj3 (exoskeleton* or orthos?s)).tw,kf.

41 exp Technology/

42 technolog*.tw,kf.

43 exp internet/

44 internet.tw,kf.

45 online.tw,kf.

46 exp computing methodologies/

47 exp Diagnosis, Computer-Assisted/

48 (computer* or computing).tw,kf.

49 exp Artificial Intelligence/

50 artificial intelligence.tw,kf.

51 AI.tw,kf.

52 machine learn*.tw,kf.

53 exp telemetry/

54 telemetry.tw,kf.

55 (telemonitor* or tele-monitor*).tw,kf.

56 Wireless Technology/

57 wireless.tw,kf.

58 exp Wearable Electronic Devices/

59 wearable?.tw,kf.

60 ((body or wrist?) adj2 (worn or wear*)).tw,kf.

61 sensor?.tw,kf.

62 sensing.tw,kf.

63 smart*.tw,kf.

64 Internet of Things/

65 internet of things.tw,kf.

66 IOT.tw,kf.

67 signal process*.tw,kf.

68 exp Signal Processing, Computer-Assisted/

69 exp Accelerometry/

70 Acceleromet*.tw,kf.

71 exp Video Recording/

72 (video* or camera*).tw,kf.

73 Inertial Measurement Unit?.tw,kf.

74 IMU.tw,kf.

75 "motion recognition".tw,kf.

76 "Real-time detection".tw,kf.

77 or/36-76

78 compensatory.tw,kf.

79 compensation*.tw,kf.

80 "non-use".tw,kf.

81 "constraint induced movement?".tw,kf.

82 or/78-81

83 ((9 and 27) or 10) and 35 and 77 and 82

84 limit 83 to english language

85 84 not (exp animals/ not exp humans/)

86 85 not ((adolescent/ or exp child/ or exp infant/) not exp adult/)

Embase Classic+Embase (Ovid)

1 cerebrovascular disease/ or exp basal ganglion hemorrhage/ or exp brain hematoma/ or exp brain hemorrhage/ or exp brain infarction/ or exp brain ischemia/ or exp carotid artery disease/ or cerebral artery disease/ or exp intracranial aneurysm/ or exp occlusive cerebrovascular disease/

2 exp cerebrovascular accident/ or stroke patient/ or stroke unit/

3 (stroke? or poststroke? or post-stroke? or cerebrovasc* or brain vasc* or cerebral vasc* or cva? or apoplex* or SAH? or TIA?).tw,kw,jx.

4 Transient Ischemic Attack*.tw,kw.

5 ((brain* or cerebr* or cerebell* or intracran* or intracerebral) adj5 (isch?emi* or infarct* or thrombo* or emboli* or occlus*)).tw,kw.

6 ((brain* or cerebr* or cerebell* or intracerebral or intracranial or subarachnoid) adj5 (haemorrhage* or hemorrhage* or haematoma* or hematoma* or bleed*)).tw,kw.

7 hemiparesis/ or hemiplegia/ or paresis/

8 (hemipleg* or hemipar* or paresis or paretic).tw,kw.

9 or/1-8

10 stroke rehabilitation/

11 rehabilitation/

12 Rehabilitation care/ or rehabilitation center/

13 Neurorehabilitation/

14 exp kinesiotherapy/

15 telerehabilitation/

16 rh.fs.

17 (rehab* or neurorehab* or telerehab*).tw,kw,jx.

18 exp physiotherapy/

19 home rehabilitation/

20 (physical adj2 therap*).tw,kw.

21 physiotherap*.tw,kw.

22 physio-therap*.tw,kw.

23 Kinesiotherap*.tw,kw.

24 exp exercise/

25 (exercise* or exercising).tw,kw.

26 ((musculoskeletal or muscle? or cardio* or cardiac* or strength* or enduranc* or balanc* or gait* or task? or coordination) adj2 (train* or retrain* or therap* or intervention*)).tw,kw.

27 or/11-26

28 exp upper limb/

29 (upper limb* or upper extremit* or upper body or arm* or shoulder* or hand* or axilla* or elbow* or forearm* or finger* or wrist*).tw,kw.

30 (dexterity or dexterous*).tw,kw.

31 exp hand strength/

32 (grip or gripping).tw,kw.

33 (grasp or grasping).tw,kw.

34 (tap or tapping).tw,kw.

35 (reach or reaching).tw,kw.

36 exp arm movement/

37 or/28-36

38 rehabilitation robot/

39 robotics/

40 robot*.tw,kw,jx.

41 automation/

42 automat*4.tw,kw.

43 (powered adj3 (exoskeleton* or orthos?s)).tw,kw.

44 robotic exoskeleton/

45 exp technology/

46 technolog*.tw,kw.

47 internet/

48 internet.tw,kw.

49 information system/ or online system/

50 online.tw,kw.

51 exp computer assisted diagnosis/

52 (computer* or computing).tw,kw.

53 exp artificial intelligence/

54 information processing/

55 artificial intelligence.tw,kw.

56 AI.tw,kw.

57 machine learn*.tw,kw.

58 exp telemetry/

59 telemetry.tw,kw.

60 (telemonitor* or tele-monitor*).tw,kw.

61 telemonitoring/

62 wireless communication/

63 wireless.tw,kw.

64 exp wearable computer/

65 wearable?.tw,kw.

66 ((body or wrist?) adj2 (worn or wear*)).tw,kw.

67 sensor?.tw,kw.

68 sensing.tw,kw.

69 smart*.tw,kw.

70 "internet of things"/

71 exp sensor/

72 signal processing/

73 signal process*.tw,kw.

74 IOT.tw,kw.

75 accelerometry/

76 accelerometer/

77 Acceleromet*.tw,kw.

78 exp videorecording/

79 (video* or camera*).tw,kw.

80 Inertial Measurement Unit?.tw,kw.

81 IMU.tw,kw.

82 "motion recognition".tw,kw.

83 "Real-time detection".tw,kw.

84 or/39-83

85 compensatory.tw,kw.

86 compensation*.tw,kw.

87 "non-use".tw,kw.

88 "constraint induced movement?".tw,kw.

89 or/85-88

90 ((9 and 27) or 10) and 37 and 84 and 89

91 (9 or 10) and 37 and 38 and 89

92 90 or 91

93 limit 92 to english language

94 93 not ((exp animals/ or exp animal experimentation/ or nonhuman/) not exp human/)

95 94 not ((exp embryo/ or exp fetus/ or exp juvenile/) not exp adult/)

96 95 not medline.cr.

Cochrane Central Register of Controlled Trials (Ovid)

1 cerebrovascular disorders/ or exp basal ganglia cerebrovascular disease/ or exp brain ischemia/ or exp carotid artery diseases/ or exp intracranial arterial diseases/ or exp intracranial arteriovenous malformations/ or exp "intracranial embolism and thrombosis"/ or exp intracranial hemorrhages/ or vasospasm, intracranial/ or vertebral artery dissection/

2 exp Stroke/

3 (stroke? or poststroke? or post-stroke? or cerebrovasc* or brain vasc* or cerebral vasc* or cva? or apoplex* or SAH? or TIA?).tw,kw,jw.

4 Transient Ischemic Attack*.tw,kw.

5 ((brain* or cerebr* or cerebell* or intracran* or intracerebral) adj5 (isch?emi* or infarct* or thrombo* or emboli* or occlus*)).tw,kw.

6 ((brain* or cerebr* or cerebell* or intracerebral or intracranial or subarachnoid) adj5 (haemorrhage* or hemorrhage* or haematoma* or hematoma* or bleed*)).tw,kw.

7 hemiplegia/ or exp paresis/

8 (hemipleg* or hemipar* or paresis or paretic).tw,kw.

9 or/1-8

10 Stroke Rehabilitation/

11 Rehabilitation/

12 Rehabilitation Centers/

13 Neurological Rehabilitation/

14 exp Exercise Therapy/

15 telerehabilitation/

16 rh.fs.

17 (rehab* or neurorehab* or telerehab*).tw,kw,jw.

18 exp Physical Therapy Modalities/

19 (physical adj2 therap*).tw,kw.

20 physiotherap*.tw,kw.

21 physio-therap*.tw,kw.

22 Kinesiotherap*.tw,kw.

23 exp Exercise/

24 exp Exercise Movement Techniques/

25 (exercise* or exercising).tw,kw.

26 ((musculoskeletal or muscle? or cardio* or cardiac* or strength* or enduranc* or balanc* or gait* or task? or coordination) adj2 (train* or retrain* or therap* or intervention*)).tw,kw.

27 or/11-26

28 exp Upper Extremity/

29 (upper limb* or upper extremit* or upper body or arm* or shoulder* or hand* or axilla* or elbow* or forearm* or finger* or wrist*).tw,kw.

30 (dexterity or dexterous*).tw,kw.

31 (grip or gripping).tw,kw.

32 (grasp or grasping).tw,kw.

33 (tap or tapping).tw,kw.

34 (reach or reaching).tw,kw.

35 or/28-34

36 Robotics/

37 robot*.tw,kw,jw.

38 Automation/

39 automat*4.tw,kw.

40 (powered adj3 (exoskeleton* or orthos?s)).tw,kw.

41 exp Technology/

42 technolog*.tw,kw.

43 exp internet/

44 internet.tw,kw.

45 online.tw,kw.

46 exp computing methodologies/

47 exp Diagnosis, Computer-Assisted/

48 (computer* or computing).tw,kw.

49 exp Artificial Intelligence/

50 artificial intelligence.tw,kw.

51 AI.tw,kw.

52 machine learn*.tw,kw.

53 exp telemetry/

54 telemetry.tw,kw.

55 (telemonitor* or tele-monitor*).tw,kw.

56 Wireless Technology/

57 wireless.tw,kw.

58 wearable?.tw,kw.

59 ((body or wrist?) adj2 (worn or wear*)).tw,kw.

60 sensor?.tw,kw.

61 sensing.tw,kw.

62 smart*.tw,kw.

63 Internet of Things/

64 internet of things.tw,kw.

65 IOT.tw,kw.

66 signal process*.tw,kw.

67 exp Signal Processing, Computer-Assisted/

68 exp Accelerometry/

69 Acceleromet*.tw,kw.

70 exp Video Recording/

71 (video* or camera*).tw,kw.

72 Inertial Measurement Unit?.tw,kw.

73 IMU.tw,kw.

74 "motion recognition".tw,kw.

75 "Real-time detection".tw,kw.

76 or/36-75

77 compensatory.tw,kw.

78 compensation*.tw,kw.

79 "non-use".tw,kw.

80 "constraint induced movement?".tw,kw.

81 or/77-80

82 ((9 and 27) or 10) and 35 and 76 and 81

83 limit 82 to english language

84 limit 82 to no language specified

85 83 or 84

86 85 not (exp animals/ not exp humans/)

87 86 not ((adolescent/ or exp child/ or exp infant/) not exp adult/)

Health Technology Assessment (Ovid)

1 cerebrovascular disorders/ or exp basal ganglia cerebrovascular disease/ or exp brain ischemia/ or exp carotid artery diseases/ or exp intracranial arterial diseases/ or exp intracranial arteriovenous malformations/ or exp "intracranial embolism and thrombosis"/ or exp intracranial hemorrhages/ or vasospasm, intracranial/ or vertebral artery dissection/

2 exp Stroke/

3 (stroke? or poststroke? or post-stroke? or cerebrovasc* or brain vasc* or cerebral vasc* or cva? or apoplex* or SAH? or TIA?).tw.

4 Transient Ischemic Attack*.tw.

5 ((brain* or cerebr* or cerebell* or intracran* or intracerebral) adj5 (isch?emi* or infarct* or thrombo* or emboli* or occlus*)).tw.

6 ((brain* or cerebr* or cerebell* or intracerebral or intracranial or subarachnoid) adj5 (haemorrhage* or hemorrhage* or haematoma* or hematoma* or bleed*)).tw.

7 hemiplegia/ or exp paresis/

8 (hemipleg* or hemipar* or paresis or paretic).tw.

9 or/1-8

10 Stroke Rehabilitation/

11 Rehabilitation/

12 Rehabilitation Centers/

13 Neurological Rehabilitation/

14 exp Exercise Therapy/

15 telerehabilitation/

16 rh.fs.

17 (rehab* or neurorehab* or telerehab*).tw.

18 exp Physical Therapy Modalities/

19 (physical adj2 therap*).tw.

20 physiotherap*.tw.

21 physio-therap*.tw.

22 Kinesiotherap*.tw.

23 exp Exercise/

24 exp Exercise Movement Techniques/

25 (exercise* or exercising).tw.

26 ((musculoskeletal or muscle? or cardio* or cardiac* or strength* or enduranc* or balanc* or gait* or task? or coordination) adj2 (train* or retrain* or therap* or intervention*)).tw.

27 or/11-26

28 exp Upper Extremity/

29 (upper limb* or upper extremit* or upper body or arm* or shoulder* or hand* or axilla* or elbow* or forearm* or finger* or wrist*).tw.

30 (dexterity or dexterous*).tw.

31 (grip or gripping).tw.

32 (grasp or grasping).tw.

33 (tap or tapping).tw.

34 (reach or reaching).tw.

35 or/28-34

36 Robotics/

37 robot*.tw.

38 Automation/

39 automat*4.tw.

40 (powered adj3 (exoskeleton* or orthos?s)).tw.

41 exp Technology/

42 technolog*.tw.

43 exp internet/

44 internet.tw.

45 online.tw.

46 exp computing methodologies/

47 exp Diagnosis, Computer-Assisted/

48 (computer* or computing).tw.

49 exp Artificial Intelligence/

50 artificial intelligence.tw.

51 AI.tw.

52 machine learn*.tw.

53 exp telemetry/

54 telemetry.tw.

55 (telemonitor* or tele-monitor*).tw.

56 Wireless Technology/

57 wireless.tw.

58 wearable?.tw.

59 ((body or wrist?) adj2 (worn or wear*)).tw.

60 sensor?.tw.

61 sensing.tw.

62 smart*.tw.

63 Internet of Things/

64 internet of things.tw.

65 IOT.tw.

66 signal process*.tw.

67 exp Signal Processing, Computer-Assisted/

68 exp Accelerometry/

69 Acceleromet*.tw.

70 exp Video Recording/

71 (video* or camera*).tw.

72 Inertial Measurement Unit?.tw.

73 IMU.tw.

74 "motion recognition".tw.

75 "Real-time detection".tw.

76 or/36-75

77 compensatory.tw.

78 compensation*.tw.

79 "non-use".tw.

80 "constraint induced movement?".tw.

81 or/77-80

82 ((9 and 27) or 10) and 35 and 76 and 81

83 limit 82 to english language

84 83 not (exp animals/ not exp humans/)

85 84 not ((adolescent/ or exp child/ or exp infant/) not exp adult/)

### SPORTDiscus (EBSCO)

| **#** | **Query** | **Limiters/Expanders** | **Last Run Via** |
| --- | --- | --- | --- |
| S1 | SO (stroke or strokes or poststroke or poststrokes or post-stroke or post-strokes or cerebrovasc* or brain vasc* or cerebral vasc* or cva or cvas or apoplex* or SAH or SAHS or TIA or TIAS) OR (stroke or strokes or poststroke or poststrokes or post-stroke or post-strokes or cerebrovasc* or brain vasc* or cerebral vasc* or cva or cvas or apoplex* or SAH or SAHS or TIA or TIAS) | Expanders - Apply equivalent subjects Search modes - Boolean/Phrase | Interface - EBSCOhost Research Databases Search Screen - Advanced Search Database - SPORTDiscus |
| S2 | Transient Ischemic Attack* | Expanders - Apply equivalent subjects Search modes - Boolean/Phrase | Interface - EBSCOhost Research Databases Search Screen - Advanced Search Database - SPORTDiscus |
| S3 | ((brain* or cerebr* or cerebell* or intracran* or intracerebral) n5 (isch#emi* or infarct* or thrombo* or emboli* or occlus*)) | Expanders - Apply equivalent subjects Search modes - Boolean/Phrase | Interface - EBSCOhost Research Databases Search Screen - Advanced Search Database - SPORTDiscus |
| S4 | ((brain* or cerebr* or cerebell* or intracerebral or intracranial or subarachnoid) n5 (haemorrhage* or hemorrhage* or haematoma* or hematoma* or bleed*)) | Expanders - Apply equivalent subjects Search modes - Boolean/Phrase | Interface - EBSCOhost Research Databases Search Screen - Advanced Search Database - SPORTDiscus |
| S5 | hemipleg* or hemipar* or paresis or paretic | Expanders - Apply equivalent subjects Search modes - Boolean/Phrase | Interface - EBSCOhost Research Databases Search Screen - Advanced Search Database - SPORTDiscus |
| S6 | ((((DE "CEREBROVASCULAR disease") OR (DE "CEREBRAL hemorrhage")) OR (DE "CEREBRAL embolism & thrombosis")) OR (DE "STROKE")) OR (DE "STROKE patients") | Expanders - Apply equivalent subjects Search modes - Boolean/Phrase | Interface - EBSCOhost Research Databases Search Screen - Advanced Search Database - SPORTDiscus |
| S7 | S1 OR S2 OR S3 OR S4 OR S5 OR S6 | Expanders - Apply equivalent subjects Search modes - Boolean/Phrase | Interface - EBSCOhost Research Databases Search Screen - Advanced Search Database - SPORTDiscus |
| S8 | DE "REHABILITATION" OR DE "MEDICAL rehabilitation" | Expanders - Apply equivalent subjects Search modes - Boolean/Phrase | Interface - EBSCOhost Research Databases Search Screen - Advanced Search Database - SPORTDiscus |
| S9 | ((DE "MOVEMENT therapy") OR (DE "EXERCISE therapy")) AND (DE "PHYSICAL therapy" OR DE "PHYSICAL therapists") | Expanders - Apply equivalent subjects Search modes - Boolean/Phrase | Interface - EBSCOhost Research Databases Search Screen - Advanced Search Database - SPORTDiscus |
| S10 | (physical n2 therap*) | Expanders - Apply equivalent subjects Search modes - Boolean/Phrase | Interface - EBSCOhost Research Databases Search Screen - Advanced Search Database - SPORTDiscus |
| S11 | SO ( rehab* or neurorehab* or telerehab* ) OR ( rehab* or neurorehab* or telerehab* ) | Expanders - Apply equivalent subjects Search modes - Boolean/Phrase | Interface - EBSCOhost Research Databases Search Screen - Advanced Search Database - SPORTDiscus |
| S12 | physiotherap* | Expanders - Apply equivalent subjects Search modes - Boolean/Phrase | Interface - EBSCOhost Research Databases Search Screen - Advanced Search Database - SPORTDiscus |
| S13 | physio-therap* | Expanders - Apply equivalent subjects Search modes - Boolean/Phrase | Interface - EBSCOhost Research Databases Search Screen - Advanced Search Database - SPORTDiscus |
| S14 | Kinesiotherap* | Expanders - Apply equivalent subjects Search modes - Boolean/Phrase | Interface - EBSCOhost Research Databases Search Screen - Advanced Search Database - SPORTDiscus |
| S15 | exercise* or exercising | Expanders - Apply equivalent subjects Search modes - Boolean/Phrase | Interface - EBSCOhost Research Databases Search Screen - Advanced Search Database - SPORTDiscus |
| S16 | ((musculoskeletal or muscle? or cardio* or cardiac* or strength* or enduranc* or balanc* or gait* or task? or coordination) n2 (train* or retrain* or therap* or intervention*)) | Expanders - Apply equivalent subjects Search modes - Boolean/Phrase | Interface - EBSCOhost Research Databases Search Screen - Advanced Search Database - SPORTDiscus |
| S17 | S8 OR S9 OR S10 OR S11 OR S12 OR S13 OR S14 OR S15 OR S16 | Expanders - Apply equivalent subjects Search modes - Boolean/Phrase | Interface - EBSCOhost Research Databases Search Screen - Advanced Search Database - SPORTDiscus |
| S18 | DE "ARM" OR DE "ELBOW" OR DE "FOREARM" OR DE "HAND" OR DE "WRIST" | Expanders - Apply equivalent subjects Search modes - Boolean/Phrase | Interface - EBSCOhost Research Databases Search Screen - Advanced Search Database - SPORTDiscus |
| S19 | (DE "GRIP strength") OR (DE "WRIST" OR DE "CARPAL bones" OR DE "WRIST extension" OR DE "WRIST flexion") | Expanders - Apply equivalent subjects Search modes - Boolean/Phrase | Interface - EBSCOhost Research Databases Search Screen - Advanced Search Database - SPORTDiscus |
| S20 | upper limb* or upper extremit* or upper body or arm* or shoulder* or hand* or axilla* or elbow* or forearm* or finger* or wrist* | Expanders - Apply equivalent subjects Search modes - Boolean/Phrase | Interface - EBSCOhost Research Databases Search Screen - Advanced Search Database - SPORTDiscus |
| S21 | dexterity or dexterous* | Expanders - Apply equivalent subjects Search modes - Boolean/Phrase | Interface - EBSCOhost Research Databases Search Screen - Advanced Search Database - SPORTDiscus |
| S22 | grip or gripping | Expanders - Apply equivalent subjects Search modes - Boolean/Phrase | Interface - EBSCOhost Research Databases Search Screen - Advanced Search Database - SPORTDiscus |
| S23 | grasp or grasping | Expanders - Apply equivalent subjects Search modes - Boolean/Phrase | Interface - EBSCOhost Research Databases Search Screen - Advanced Search Database - SPORTDiscus |
| S24 | tap or tapping | Expanders - Apply equivalent subjects Search modes - Boolean/Phrase | Interface - EBSCOhost Research Databases Search Screen - Advanced Search Database - SPORTDiscus |
| S25 | reach or reaching | Expanders - Apply equivalent subjects Search modes - Boolean/Phrase | Interface - EBSCOhost Research Databases Search Screen - Advanced Search Database - SPORTDiscus |
| S26 | S18 OR S19 OR S20 OR S21 OR S22 OR S23 OR S24 OR S25 | Expanders - Apply equivalent subjects Search modes - Boolean/Phrase | Interface - EBSCOhost Research Databases Search Screen - Advanced Search Database - SPORTDiscus |
| S27 | DE "ROBOTICS in sports" | Expanders - Apply equivalent subjects Search modes - Boolean/Phrase | Interface - EBSCOhost Research Databases Search Screen - Advanced Search Database - SPORTDiscus |
| S28 | robot* OR SO robot* | Expanders - Apply equivalent subjects Search modes - Boolean/Phrase | Interface - EBSCOhost Research Databases Search Screen - Advanced Search Database - SPORTDiscus |
| S29 | automat* | Expanders - Apply equivalent subjects Search modes - Boolean/Phrase | Interface - EBSCOhost Research Databases Search Screen - Advanced Search Database - SPORTDiscus |
| S30 | (powered n3 (exoskeleton* or orthos#s)) | Expanders - Apply equivalent subjects Search modes - Boolean/Phrase | Interface - EBSCOhost Research Databases Search Screen - Advanced Search Database - SPORTDiscus |
| S31 | technolog* | Expanders - Apply equivalent subjects Search modes - Boolean/Phrase | Interface - EBSCOhost Research Databases Search Screen - Advanced Search Database - SPORTDiscus |
| S32 | DE "INTERNET" | Expanders - Apply equivalent subjects Search modes - Boolean/Phrase | Interface - EBSCOhost Research Databases Search Screen - Advanced Search Database - SPORTDiscus |
| S33 | internet | Expanders - Apply equivalent subjects Search modes - Boolean/Phrase | Interface - EBSCOhost Research Databases Search Screen - Advanced Search Database - SPORTDiscus |
| S34 | online | Expanders - Apply equivalent subjects Search modes - Boolean/Phrase | Interface - EBSCOhost Research Databases Search Screen - Advanced Search Database - SPORTDiscus |
| S35 | computer* or computing | Expanders - Apply equivalent subjects Search modes - Boolean/Phrase | Interface - EBSCOhost Research Databases Search Screen - Advanced Search Database - SPORTDiscus |
| S36 | artificial intelligence | Expanders - Apply equivalent subjects Search modes - Boolean/Phrase | Interface - EBSCOhost Research Databases Search Screen - Advanced Search Database - SPORTDiscus |
| S37 | AI | Expanders - Apply equivalent subjects Search modes - Boolean/Phrase | Interface - EBSCOhost Research Databases Search Screen - Advanced Search Database - SPORTDiscus |
| S38 | machine learn* | Expanders - Apply equivalent subjects Search modes - Boolean/Phrase | Interface - EBSCOhost Research Databases Search Screen - Advanced Search Database - SPORTDiscus |
| S39 | telemetry | Expanders - Apply equivalent subjects Search modes - Boolean/Phrase | Interface - EBSCOhost Research Databases Search Screen - Advanced Search Database - SPORTDiscus |
| S40 | (telemonitor* or tele-monitor*) | Expanders - Apply equivalent subjects Search modes - Boolean/Phrase | Interface - EBSCOhost Research Databases Search Screen - Advanced Search Database - SPORTDiscus |
| S41 | wireless | Expanders - Apply equivalent subjects Search modes - Boolean/Phrase | Interface - EBSCOhost Research Databases Search Screen - Advanced Search Database - SPORTDiscus |
| S42 | wearable or wearables | Expanders - Apply equivalent subjects Search modes - Boolean/Phrase | Interface - EBSCOhost Research Databases Search Screen - Advanced Search Database - SPORTDiscus |
| S43 | ((body or wrist?) n2 (worn or wear*)) | Expanders - Apply equivalent subjects Search modes - Boolean/Phrase | Interface - EBSCOhost Research Databases Search Screen - Advanced Search Database - SPORTDiscus |
| S44 | sensor or sensors | Expanders - Apply equivalent subjects Search modes - Boolean/Phrase | Interface - EBSCOhost Research Databases Search Screen - Advanced Search Database - SPORTDiscus |
| S45 | sensing | Expanders - Apply equivalent subjects Search modes - Boolean/Phrase | Interface - EBSCOhost Research Databases Search Screen - Advanced Search Database - SPORTDiscus |
| S46 | smart* | Expanders - Apply equivalent subjects Search modes - Boolean/Phrase | Interface - EBSCOhost Research Databases Search Screen - Advanced Search Database - SPORTDiscus |
| S47 | internet of things | Expanders - Apply equivalent subjects Search modes - Boolean/Phrase | Interface - EBSCOhost Research Databases Search Screen - Advanced Search Database - SPORTDiscus |
| S48 | IOT | Expanders - Apply equivalent subjects Search modes - Boolean/Phrase | Interface - EBSCOhost Research Databases Search Screen - Advanced Search Database - SPORTDiscus |
| S49 | signal process* | Expanders - Apply equivalent subjects Search modes - Boolean/Phrase | Interface - EBSCOhost Research Databases Search Screen - Advanced Search Database - SPORTDiscus |
| S50 | (video* or camera*) | Expanders - Apply equivalent subjects Search modes - Boolean/Phrase | Interface - EBSCOhost Research Databases Search Screen - Advanced Search Database - SPORTDiscus |
| S51 | Inertial Measurement Unit OR Inertial Measurement Units | Expanders - Apply equivalent subjects Search modes - Boolean/Phrase | Interface - EBSCOhost Research Databases Search Screen - Advanced Search Database - SPORTDiscus |
| S52 | IMU | Expanders - Apply equivalent subjects Search modes - Boolean/Phrase | Interface - EBSCOhost Research Databases Search Screen - Advanced Search Database - SPORTDiscus |
| S53 | "motion recognition" | Expanders - Apply equivalent subjects Search modes - Boolean/Phrase | Interface - EBSCOhost Research Databases Search Screen - Advanced Search Database - SPORTDiscus |
| S54 | "Real-time detection" | Expanders - Apply equivalent subjects Search modes - Boolean/Phrase | Interface - EBSCOhost Research Databases Search Screen - Advanced Search Database - SPORTDiscus |
| S55 | S27 OR S28 OR S29 OR S30 OR S31 OR S32 OR S33 OR S34 OR S35 OR S36 OR S37 OR S38 OR S39 OR S40 OR S41 OR S42 OR S43 OR S44 OR S45 OR S46 OR S47 OR S48 OR S49 OR S50 OR S51 OR S52 OR S53 OR S54 | Expanders - Apply equivalent subjects Search modes - Boolean/Phrase | Interface - EBSCOhost Research Databases Search Screen - Advanced Search Database - SPORTDiscus |
| S56 | compensatory | Expanders - Apply equivalent subjects Search modes - Boolean/Phrase | Interface - EBSCOhost Research Databases Search Screen - Advanced Search Database - SPORTDiscus |
| S57 | compensation* | Expanders - Apply equivalent subjects Search modes - Boolean/Phrase | Interface - EBSCOhost Research Databases Search Screen - Advanced Search Database - SPORTDiscus |
| S58 | "non-use" | Expanders - Apply equivalent subjects Search modes - Boolean/Phrase | Interface - EBSCOhost Research Databases Search Screen - Advanced Search Database - SPORTDiscus |
| S59 | "constraint induced movement?" | Expanders - Apply equivalent subjects Search modes - Boolean/Phrase | Interface - EBSCOhost Research Databases Search Screen - Advanced Search Database - SPORTDiscus |
| S60 | S56 OR S57 OR S58 OR S59 | Expanders - Apply equivalent subjects Search modes - Boolean/Phrase | Interface - EBSCOhost Research Databases Search Screen - Advanced Search Database - SPORTDiscus |
| S61 | S7 AND S17 AND S26 AND S55 AND S60 | Limiters - Language: English Expanders - Apply equivalent subjects Search modes - Boolean/Phrase | Interface - EBSCOhost Research Databases Search Screen - Advanced Search Database - SPORTDiscus |

### Scopus

(((TITLE-ABS-KEY((stroke? or poststroke? or post-stroke? or cerebrovasc* or brain vasc* or cerebral vasc* or cva? or apoplex* or SAH? or TIA?)) OR SRCTITLE((stroke? or poststroke? or post-stroke? or cerebrovasc* or brain vasc* or cerebral vasc* or cva? or apoplex* or SAH? or TIA?)))) OR (TITLE-ABS-KEY(Transient Ischemic Attack*)) OR (TITLE-ABS-KEY(((brain* or cerebr* or cerebell* or intracran* or intracerebral) W/5 (isch?emi* or infarct* or thrombo* or emboli* or occlus*)))) OR (TITLE-ABS-KEY(((brain* or cerebr* or cerebell* or intracerebral or intracranial or subarachnoid) W/5 (haemorrhage* or hemorrhage* or haematoma* or hematoma* or bleed*)))) OR (TITLE-ABS-KEY(hemipleg* or hemipar* or paresis or paretic))) and (((TITLE-ABS-KEY((rehab* or neurorehab* or telerehab*)) OR SRCTITLE((rehab* or neurorehab* or telerehab*) ))) OR (TITLE-ABS-KEY(physical W/2 therap*)) OR (TITLE-ABS-KEY(physiotherap*)) OR (TITLE-ABS-KEY(physio-therap*)) OR (TITLE-ABS-KEY(Kinesiotherap*)) OR (TITLE-ABS-KEY(exercise* or exercising)) OR (TITLE-ABS-KEY(((musculoskeletal or muscle? or cardio* or cardiac* or strength* or enduranc* or balanc* or gait* or task? or coordination) W/2 (train* or retrain* or therap* or intervention*))))) and ((TITLE-ABS-KEY(upper limb* or upper extremit* or upper body or arm* or shoulder* or hand* or axilla* or elbow* or forearm* or finger* or wrist*) OR TITLE-ABS-KEY(dexterity or dexterous*) OR TITLE-ABS-KEY(grip or gripping) OR TITLE-ABS-KEY(grasp or grasping) OR TITLE-ABS-KEY(tap or tapping) OR TITLE-ABS-KEY(reach or reaching))) and (((TITLE-ABS-KEY(robot*) OR SRCTITLE(robot*))) or (TITLE-ABS-KEY(automat*)) OR (TITLE-ABS-KEY((powered W/3 (exoskeleton* or orthos?s)))) OR (TITLE-ABS-KEY(technolog*)) OR (TITLE-ABS-KEY(internet)) OR (TITLE-ABS-KEY(internet)) OR (TITLE-ABS-KEY(computer* or computing)) OR (TITLE-ABS-KEY(AI)) OR (TITLE-ABS-KEY(telemetry)) OR (TITLE-ABS-KEY((telemonitor* or tele-monitor*))) OR (TITLE-ABS-KEY(wireless)) OR (TITLE-ABS-KEY(wearable or wearables)) OR (TITLE-ABS-KEY(((body or wrist?) W/2 (worn or wear*)))) OR (TITLE-ABS-KEY(sensor or sensors)) OR (TITLE-ABS-KEY(sensing)) OR (TITLE-ABS-KEY(smart*)) OR (TITLE-ABS-KEY(IOT)) OR (TITLE-ABS-KEY("signal process*")) OR (TITLE-ABS-KEY("internet of things")) OR (TITLE-ABS-KEY("machine learn*")) OR (TITLE-ABS-KEY("artificial intelligence")) Or (TITLE-ABS-KEY(Acceleromet*)) OR (TITLE-ABS-KEY(video* or camera*)) OR (TITLE-ABS-KEY("Inertial Measurement Unit?")) OR (TITLE-ABS-KEY(IMU)) or (TITLE-ABS-KEY("motion recognition")) Or (TITLE-ABS-KEY("Real-time detection"))) and ((TITLE-ABS-KEY(compensatory) OR TITLE-ABS-KEY(compensation*) OR TITLE-ABS-KEY("non-use") OR TITLE-ABS-KEY("constraint induced movement?"))) AND ORIG-LOAD-DATE > 20200519 AND ( LIMIT-TO ( LANGUAGE,"English" ) )

###

### Compendex (Engineering Village)

( ((({COMPENSATION}) WN CV) OR (({COMPENSATION METHOD}) WN CV) OR (({COMPENSATION METHODS}) WN CV) OR ((compensation) WN KY) OR ((compensatory) WN KY) OR (({non-use}) WN KY) OR (({constraint induced movement} OR {constraint induced movements}) WN KY)) AND (1884-2020 WN YR)) AND ( ((((({ROBOTICS}) WN CV) OR (({TECHNOLOGY}) WN CV) OR (({AUTOMATION}) WN CV) OR (({INTERNET OF THINGS}) WN CV) OR (({INTERNET}) WN CV) OR (({ONLINE AGE}) WN CV) OR (({COMPUTER}) WN CV) OR (({AI}) WN CV) OR (({WEARABLE COMPUTERS}) WN CV) OR (({WEARABLE SENSORS}) WN CV) OR (({WEARABLE TECHNOLOGY}) WN CV) OR (({EXOSKELETON (ROBOTICS)}) WN CV) OR (({SENSORS}) WN CV) OR (({MACHINE LEARNING}) WN CV) OR (({ACCELEROMETER}) WN CV) OR (({IMU}) WN CV) OR ((robot*) WN KY) OR ((robot*) WN ST) OR ((automat*) WN KY) OR ((powered NEAR/3 exoskeleton*) WN KY) OR ((powered NEAR/3 orthos?s) WN KY) OR ((technolog* OR internet OR online OR computer OR computing OR AI OR {artificial intelligence} OR {machine learning} OR telemetry OR telemonitor* OR {tele-monitoring} OR {telemonitor} OR wireless OR wearable OR wearables OR sensor OR sensors OR sensing OR smart* OR IOT OR {signal processing} OR video* OR camera* OR {Inertial Measurement Unit} OR {Inertial Measurement Units} OR IMU OR {motion recognition} OR {real-time detection}) WN KY) OR ((body NEAR/2 worn) WN KY) OR ((body NEAR/2 wear) WN KY) OR ((wrist NEAR/2 worn) WN KY) OR ((wrist NEAR/2 wear) WN KY)) AND (1884-2020 WN YR)) AND (((((({upper limb} OR {upper limbs} OR {upper extremity} OR {upper extremities} OR {upper body} OR arm* OR shoulder* OR hand* OR axilla* OR elbow* OR forearm* OR finger* OR wrist*) WN KY) OR ((dexterity OR dexterous* OR grip OR gripping OR grasp OR grasping OR tap OR tapping OR reach OR reaching) WN KY)) AND (1884-2020 WN YR)) AND (((((({STROKE PATIENTS}) WN CV) OR ((stroke OR strokes OR poststroke OR poststrokes OR {post-stroke} OR {post-strokes} OR cerebrovasc* OR brain vasc* OR cerebral vasc* OR cva OR cvas OR apoplex* OR SAH OR SAHs OR TIA OR TIAs) WN KY) OR ((stroke OR strokes OR poststroke OR poststrokes OR {post-stroke} OR {post-strokes} OR cerebrovasc* OR brain vasc* OR cerebral vasc* OR cva OR cvas OR apoplex* OR SAH OR SAHs OR TIA OR TIAs) WN ST) OR (({Transient Ischemic Attack} OR {Transient Ischemic Attacks}) WN KY) OR ((brain* NEAR/5 isch*emi*) WN KY) OR ((brain* NEAR/5 infarct*) WN KY) OR ((brain* NEAR/5 thrombo*) WN KY) OR ((brain* NEAR/5 emboli*) WN KY) OR ((brain* NEAR/5 occlus*) WN KY) OR ((cerebr* NEAR/5 isch*emi*) WN KY) OR ((cerebr* NEAR/5 infarct*) WN KY) OR ((cerebr* NEAR/5 thrombo*) WN KY) OR ((cerebr* NEAR/5 emboli*) WN KY) OR ((cerebr* NEAR/5 occlus*) WN KY) OR ((cerebell* NEAR/5 isch*emi*) WN KY) OR ((cerebell* NEAR/5 infarct*) WN KY) OR ((cerebell* NEAR/5 thrombo*) WN KY) OR ((cerebell* NEAR/5 emboli*) WN KY) OR ((cerebell* NEAR/5 occlus*) WN KY) OR ((intracran* NEAR/5 isch*emi*) WN KY) OR ((intracran* NEAR/5 infarct*) WN KY) OR ((intracran* NEAR/5 thrombo*) WN KY) OR ((intracran* NEAR/5 emboli*) WN KY) OR ((intracran* NEAR/5 occlus*) WN KY) OR ((intracerebral* NEAR/5 isch*emi*) WN KY) OR ((intracerebral* NEAR/5 infarct*) WN KY) OR ((intracerebral* NEAR/5 thrombo*) WN KY) OR ((intracerebral* NEAR/5 emboli*) WN KY) OR ((intracerebral* NEAR/5 occlus*) WN KY) OR ((brain* NEAR/5 haemorrhage*) WN KY) OR ((brain* NEAR/5 hemorrhage*) WN KY) OR ((brain* NEAR/5 haematoma*) WN KY) OR ((brain* NEAR/5 hematoma*) WN KY) OR ((brain* NEAR/5 bleed*) WN KY) OR ((cerebr* NEAR/5 haemorrhage*) WN KY) OR ((cerebr* NEAR/5 hemorrhage*) WN KY) OR ((cerebr* NEAR/5 haematoma*) WN KY) OR ((cerebr* NEAR/5 hematoma*) WN KY) OR ((cerebr* NEAR/5 bleed*) WN KY) OR ((cerebell* NEAR/5 haemorrhage*) WN KY) OR ((cerebell* NEAR/5 hemorrhage*) WN KY) OR ((cerebell* NEAR/5 haematoma*) WN KY) OR ((cerebell* NEAR/5 hematoma*) WN KY) OR ((cerebell* NEAR/5 bleed*) WN KY) OR ((intracerebral* NEAR/5 haemorrhage*) WN KY) OR ((intracerebral* NEAR/5 hemorrhage*) WN KY) OR ((intracerebral* NEAR/5 haematoma*) WN KY) OR ((intracerebral* NEAR/5 hematoma*) WN KY) OR ((intracerebral* NEAR/5 bleed*) WN KY) OR ((intracranial* NEAR/5 haemorrhage*) WN KY) OR ((intracranial* NEAR/5 hemorrhage*) WN KY) OR ((intracranial* NEAR/5 haematoma*) WN KY) OR ((intracranial* NEAR/5 hematoma*) WN KY) OR ((intracranial* NEAR/5 bleed*) WN KY) OR ((subarachnoid* NEAR/5 haemorrhage*) WN KY) OR ((subarachnoid* NEAR/5 hemorrhage*) WN KY) OR ((subarachnoid* NEAR/5 haematoma*) WN KY) OR ((subarachnoid* NEAR/5 hematoma*) WN KY) OR ((subarachnoid* NEAR/5 bleed*) WN KY) OR ((hemipleg* OR hemipar* OR paresis OR paretic) WN KY)) AND (1884-2020 WN YR)) AND (((({REHABILITATION}) WN CV) OR (({REHABILITATION ENGINEERING}) WN CV) OR (({REHABILITATION EQUIPMENT}) WN CV) OR (({REHABILITATION TECHNIQUES}) WN CV) OR (({EXERCISE}) WN CV) OR (({EXERCISE EQUIPMENT}) WN CV) OR ((*rehab*) WN KY) OR ((*rehab*) WN ST) OR ((physical NEAR/2 therap*) WN KY) OR ((physiotherap*) WN KY) OR ((physio-therap*) WN KY) OR ((Kinesiotherap*) WN KY) OR ((exercise* OR exercising) WN KY) OR ((musculoskeletal NEAR/2 train*) WN KY) OR ((musculoskeletal NEAR/2 retrain*) WN KY) OR ((musculoskeletal NEAR/2 therap*) WN KY) OR ((musculoskeletal NEAR/2 intervention*) WN KY) OR ((muscle? NEAR/2 train*) WN KY) OR ((muscle? NEAR/2 retrain*) WN KY) OR ((muscle? NEAR/2 therap*) WN KY) OR ((muscle? NEAR/2 intervention*) WN KY) OR ((cardio* NEAR/2 train*) WN KY) OR ((cardio* NEAR/2 retrain*) WN KY) OR ((cardio* NEAR/2 therap*) WN KY) OR ((cardio* NEAR/2 intervention*) WN KY) OR ((cardiac* NEAR/2 train*) WN KY) OR ((cardiac* NEAR/2 retrain*) WN KY) OR ((cardiac* NEAR/2 therap*) WN KY) OR ((cardiac* NEAR/2 intervention*) WN KY) OR ((strength* NEAR/2 train*) WN KY) OR ((strength* NEAR/2 retrain*) WN KY) OR ((strength* NEAR/2 therap*) WN KY) OR ((strength* NEAR/2 intervention*) WN KY) OR ((enduranc* NEAR/2 train*) WN KY) OR ((enduranc* NEAR/2 retrain*) WN KY) OR ((enduranc* NEAR/2 therap*) WN KY) OR ((enduranc* NEAR/2 intervention*) WN KY) OR ((balanc* NEAR/2 train*) WN KY) OR ((balanc* NEAR/2 retrain*) WN KY) OR ((balanc* NEAR/2 therap*) WN KY) OR ((balanc* NEAR/2 intervention*) WN KY) OR ((gait* NEAR/2 train*) WN KY) OR ((gait* NEAR/2 retrain*) WN KY) OR ((gait* NEAR/2 therap*) WN KY) OR ((gait* NEAR/2 intervention*) WN KY) OR ((task? NEAR/2 train*) WN KY) OR ((task? NEAR/2 retrain*) WN KY) OR ((task? NEAR/2 therap*) WN KY) OR ((task? NEAR/2 intervention*) WN KY) OR ((coordination NEAR/2 train*) WN KY) OR ((coordination NEAR/2 retrain*) WN KY) OR ((coordination NEAR/2 therap*) WN KY) OR ((coordination NEAR/2 intervention*) WN KY))))))))) AND ({english} WN LA)

### INSPEC (Engineering Village)

( (((stroke OR strokes OR poststroke OR poststrokes OR {post-stroke} OR {post-strokes} OR cerebrovasc* OR brain vasc* OR cerebral vasc* OR cva OR cvas OR apoplex* OR SAH OR SAHs OR TIA OR TIAs) WN KY) OR ((stroke OR strokes OR poststroke OR poststrokes OR {post-stroke} OR {post-strokes} OR cerebrovasc* OR brain vasc* OR cerebral vasc* OR cva OR cvas OR apoplex* OR SAH OR SAHs OR TIA OR TIAs) WN ST) OR (({Transient Ischemic Attack} OR {Transient Ischemic Attacks}) WN KY) OR ((brain* NEAR/5 isch*emi*) WN KY) OR ((brain* NEAR/5 infarct*) WN KY) OR ((brain* NEAR/5 thrombo*) WN KY) OR ((brain* NEAR/5 emboli*) WN KY) OR ((brain* NEAR/5 occlus*) WN KY) OR ((cerebr* NEAR/5 isch*emi*) WN KY) OR ((cerebr* NEAR/5 infarct*) WN KY) OR ((cerebr* NEAR/5 thrombo*) WN KY) OR ((cerebr* NEAR/5 emboli*) WN KY) OR ((cerebr* NEAR/5 occlus*) WN KY) OR ((cerebell* NEAR/5 isch*emi*) WN KY) OR ((cerebell* NEAR/5 infarct*) WN KY) OR ((cerebell* NEAR/5 thrombo*) WN KY) OR ((cerebell* NEAR/5 emboli*) WN KY) OR ((cerebell* NEAR/5 occlus*) WN KY) OR ((intracran* NEAR/5 isch*emi*) WN KY) OR ((intracran* NEAR/5 infarct*) WN KY) OR ((intracran* NEAR/5 thrombo*) WN KY) OR ((intracran* NEAR/5 emboli*) WN KY) OR ((intracran* NEAR/5 occlus*) WN KY) OR ((intracerebral* NEAR/5 isch*emi*) WN KY) OR ((intracerebral* NEAR/5 infarct*) WN KY) OR ((intracerebral* NEAR/5 thrombo*) WN KY) OR ((intracerebral* NEAR/5 emboli*) WN KY) OR ((intracerebral* NEAR/5 occlus*) WN KY) OR ((brain* NEAR/5 haemorrhage*) WN KY) OR ((brain* NEAR/5 hemorrhage*) WN KY) OR ((brain* NEAR/5 haematoma*) WN KY) OR ((brain* NEAR/5 hematoma*) WN KY) OR ((brain* NEAR/5 bleed*) WN KY) OR ((cerebr* NEAR/5 haemorrhage*) WN KY) OR ((cerebr* NEAR/5 hemorrhage*) WN KY) OR ((cerebr* NEAR/5 haematoma*) WN KY) OR ((cerebr* NEAR/5 hematoma*) WN KY) OR ((cerebr* NEAR/5 bleed*) WN KY) OR ((cerebell* NEAR/5 haemorrhage*) WN KY) OR ((cerebell* NEAR/5 hemorrhage*) WN KY) OR ((cerebell* NEAR/5 haematoma*) WN KY) OR ((cerebell* NEAR/5 hematoma*) WN KY) OR ((cerebell* NEAR/5 bleed*) WN KY) OR ((intracerebral* NEAR/5 haemorrhage*) WN KY) OR ((intracerebral* NEAR/5 hemorrhage*) WN KY) OR ((intracerebral* NEAR/5 haematoma*) WN KY) OR ((intracerebral* NEAR/5 hematoma*) WN KY) OR ((intracerebral* NEAR/5 bleed*) WN KY) OR ((intracranial* NEAR/5 haemorrhage*) WN KY) OR ((intracranial* NEAR/5 hemorrhage*) WN KY) OR ((intracranial* NEAR/5 haematoma*) WN KY) OR ((intracranial* NEAR/5 hematoma*) WN KY) OR ((intracranial* NEAR/5 bleed*) WN KY) OR ((subarachnoid* NEAR/5 haemorrhage*) WN KY) OR ((subarachnoid* NEAR/5 hemorrhage*) WN KY) OR ((subarachnoid* NEAR/5 haematoma*) WN KY) OR ((subarachnoid* NEAR/5 hematoma*) WN KY) OR ((subarachnoid* NEAR/5 bleed*) WN KY) OR ((hemipleg* OR hemipar* OR paresis OR paretic) WN KY)) AND (1896-2020 WN YR)) AND ( (((rehab*) WN KY) OR ((rehab*) WN ST) OR ((physical NEAR/2 therap*) WN KY) OR ((physiotherap*) WN KY) OR ((physio-therap*) WN KY) OR ((Kinesiotherap*) WN KY) OR ((exercise* OR exercising) WN KY) OR ((musculoskeletal NEAR/2 train*) WN KY) OR ((musculoskeletal NEAR/2 retrain*) WN KY) OR ((musculoskeletal NEAR/2 therap*) WN KY) OR ((musculoskeletal NEAR/2 intervention*) WN KY) OR ((muscle? NEAR/2 train*) WN KY) OR ((muscle? NEAR/2 retrain*) WN KY) OR ((muscle? NEAR/2 therap*) WN KY) OR ((muscle? NEAR/2 intervention*) WN KY) OR ((cardio* NEAR/2 train*) WN KY) OR ((cardio* NEAR/2 retrain*) WN KY) OR ((cardio* NEAR/2 therap*) WN KY) OR ((cardio* NEAR/2 intervention*) WN KY) OR ((cardiac* NEAR/2 train*) WN KY) OR ((cardiac* NEAR/2 retrain*) WN KY) OR ((cardiac* NEAR/2 therap*) WN KY) OR ((cardiac* NEAR/2 intervention*) WN KY) OR ((strength* NEAR/2 train*) WN KY) OR ((strength* NEAR/2 retrain*) WN KY) OR ((strength* NEAR/2 therap*) WN KY) OR ((strength* NEAR/2 intervention*) WN KY) OR ((enduranc* NEAR/2 train*) WN KY) OR ((enduranc* NEAR/2 retrain*) WN KY) OR ((enduranc* NEAR/2 therap*) WN KY) OR ((enduranc* NEAR/2 intervention*) WN KY) OR ((balanc* NEAR/2 train*) WN KY) OR ((balanc* NEAR/2 retrain*) WN KY) OR ((balanc* NEAR/2 therap*) WN KY) OR ((balanc* NEAR/2 intervention*) WN KY) OR ((gait* NEAR/2 train*) WN KY) OR ((gait* NEAR/2 retrain*) WN KY) OR ((gait* NEAR/2 therap*) WN KY) OR ((gait* NEAR/2 intervention*) WN KY) OR ((task? NEAR/2 train*) WN KY) OR ((task? NEAR/2 retrain*) WN KY) OR ((task? NEAR/2 therap*) WN KY) OR ((task? NEAR/2 intervention*) WN KY) OR ((coordination NEAR/2 train*) WN KY) OR ((coordination NEAR/2 retrain*) WN KY) OR ((coordination NEAR/2 therap*) WN KY) OR ((coordination NEAR/2 intervention*) WN KY)) AND (1896-2020 WN YR)) AND ( ((({upper limb} OR {upper limbs} OR {upper extremity} OR {upper extremities} OR {upper body} OR arm* OR shoulder* OR hand* OR axilla* OR elbow* OR forearm* OR finger* OR wrist*) WN KY) OR ((dexterity OR dexterous* OR grip OR gripping OR grasp OR grasping OR tap OR tapping OR reach OR reaching) WN KY)) AND (1896-2020 WN YR)) AND ( ((({ROBOTS}) WN CV) OR (({TECHNOLOGY}) WN CV) OR (({INTERNET}) WN CV) OR (({INTERNET OF THINGS}) WN CV) OR (({ARTIFICIAL INTELLIGENCE}) WN CV) OR (({COMPUTER AIDED ANALYSIS}) WN CV) OR (({COMPUTER ANIMATION}) WN CV) OR (({COMPUTERS}) WN CV) OR (({SENSORS}) WN CV) OR ((robot*) WN KY) OR ((robot*) WN ST) OR ((automat*) WN KY) OR ((powered NEAR/3 exoskeleton*) WN KY) OR ((powered NEAR/3 orthos?s) WN KY) OR ((technolog* OR internet OR online OR computer OR computing OR AI OR {artificial intelligence} OR {machine learning} OR telemetry OR telemonitor* OR {tele-monitoring} OR {telemonitor} OR wireless OR wearable OR wearables OR sensor OR sensors OR sensing OR smart* OR IOT OR {signal processing} OR video* OR camera* OR {Inertial Measurement Unit} OR {Inertial Measurement Units} OR IMU OR {motion recognition} OR {real-time detection}) WN KY) OR ((body NEAR/2 worn) WN KY) OR ((body NEAR/2 wear) WN KY) OR ((wrist NEAR/2 worn) WN KY) OR ((wrist NEAR/2 wear) WN KY)) AND (1896-2020 WN YR)) AND ( ((({COMPENSATION}) WN CV) OR ((compensation) WN KY) OR ((compensatory) WN KY) OR (({non-use}) WN KY) OR (({constraint induced movement} OR {constraint induced movements}) WN KY)) AND (1896-2020 WN YR)) AND ({english} WN LA)

### ACM Digital Library

(Title:(stroke OR strokes OR poststroke OR poststrokes OR "post-stroke" OR "post-strokes" OR cerebrovasc* OR brain vasc* OR cerebral vasc* OR cva OR cvas OR apoplex* OR SAH OR SAHs OR TIA OR TIAs OR "Transient Ischemic Attack" OR "Transient Ischemic Attacks") OR Abstract:(stroke OR strokes OR poststroke OR poststrokes OR "post-stroke" OR "post-strokes" OR cerebrovasc* OR brain vasc* OR cerebral vasc* OR cva OR cvas OR apoplex* OR SAH OR SAHs OR TIA OR TIAs OR "Transient Ischemic Attack" OR "Transient Ischemic Attacks")) AND (Title:(rehab* OR neurorehab* OR telerehab* OR physiotherap* OR "physio-therapy" OR "physio-therapist" OR "physical therapy" OR "physical therapist" OR Kinesiotherap* OR exercise* OR exercising) OR Abstract:(rehab* OR neurorehab* OR telerehab* OR physiotherap* OR "physio-therapy" OR "physio-therapist" OR "physical therapy" OR "physical therapist" OR Kinesiotherap* OR exercise* OR exercising)) AND (Title:("upper limb" OR "upper limbs" OR "upper extremity" OR "upper extremities" OR "upper body" OR arm* OR shoulder* OR hand* OR axilla* OR elbow* OR forearm* OR finger* OR wrist* OR dexterity OR dexterous* OR grip OR gripping OR grasp OR grasping OR tap OR tapping OR reach OR reaching) OR Abstract:("upper limb" OR "upper limbs" OR "upper extremity" OR "upper extremities" OR "upper body" OR arm* OR shoulder* OR hand* OR axilla* OR elbow* OR forearm* OR finger* OR wrist* OR dexterity OR dexterous* OR grip OR gripping OR grasp OR grasping OR tap OR tapping OR reach OR reaching)) AND (Title:(robot* OR automat* OR technolog* OR internet OR online OR computer OR computing OR AI OR "artificial intelligence" OR "machine learning" OR telemetry OR telemonitor* OR "tele-monitoring" OR "telemonitor" OR wireless OR wearable OR wearables OR sensor OR sensors OR sensing OR smart* OR IOT OR "signal processing" OR video* OR camera* OR "Inertial Measurement Unit" OR "Inertial Measurement Units" OR IMU OR "motion recognition" OR "real-time detection") OR Abstract:(robot* OR automat* OR technolog* OR internet OR online OR computer OR computing OR AI OR "artificial intelligence" OR "machine learning" OR telemetry OR telemonitor* OR "tele-monitoring" OR "telemonitor" OR wireless OR wearable OR wearables OR sensor OR sensors OR sensing OR smart* OR IOT OR "signal processing" OR video* OR camera* OR "Inertial Measurement Unit" OR "Inertial Measurement Units" OR IMU OR "motion recognition" OR "real-time detection")) AND (Title:(compensation OR compensatory OR "non-use" OR "constraint induced movement" OR "constraint induced movements") OR Abstract:(compensation OR compensatory OR "non-use" OR "constraint induced movement" OR "constraint induced movements"))

### IEEE Xplore

(("Document Title":stroke OR strokes OR poststroke OR poststrokes OR "post-stroke" OR "post-strokes" OR cerebrovascular OR brain vascucal OR cerebral vascular OR cva OR cvas OR apoplexy OR SAH OR SAHs OR TIA OR TIAs OR "Transient Ischemic Attack" OR "Transient Ischemic Attacks") OR ("Abstract":stroke OR strokes OR poststroke OR poststrokes OR "post-stroke" OR "post-strokes" OR cerebrovascular OR brain vascucal OR cerebral vascular OR cva OR cvas OR apoplexy OR SAH OR SAHs OR TIA OR TIAs OR "Transient Ischemic Attack" OR "Transient Ischemic Attacks")) AND (("Document Title":rehab or rehabilitation OR neurorehab or neurorehabilitation OR telerehab or telerehabilitation OR physiotherapy or physiotherapist or physiotherapists OR "physio-therapy" OR "physio-therapist" OR "physical therapy" OR "physical therapist" OR Kinesiotherapy or kinesiotherapist or kinesiotherapists OR exercise OR exercises OR exercising) OR ("Abstract":rehab or rehabilitation OR neurorehab or neurorehabilitation OR telerehab or telerehabilitation OR physiotherapy or physiotherapist or physiotherapists OR "physio-therapy" OR "physio-therapist" OR "physical therapy" OR "physical therapist" OR Kinesiotherapy or kinesiotherapist or kinesiotherapists OR exercise OR exercises OR exercising)) AND (("Document Title":"upper limb" OR "upper limbs" OR "upper extremity" OR "upper extremities" OR "upper body" OR arm or arms OR shoulder or shoulders OR hand or hands OR axilla or axillas OR elbow or elbows OR forearm or forearms OR finger or fingers OR wrist or wrists OR dexterity OR dexterous OR grip OR gripping OR grasp OR grasping OR tap OR tapping OR reach OR reaching) OR ("Abstract":"upper limb" OR "upper limbs" OR "upper extremity" OR "upper extremities" OR "upper body" OR arm or arms OR shoulder or shoulders OR hand or hands OR axilla or axillas OR elbow or elbows OR forearm or forearms OR finger or fingers OR wrist or wrists OR dexterity OR dexterous OR grip OR gripping OR grasp OR grasping OR tap OR tapping OR reach OR reaching)) AND (("Document Title": robot* or automation OR automated or technology or technological or technologies OR internet OR online OR computer OR computing OR AI OR "artificial intelligence" OR "machine learning" OR telemetry OR telemonitoring or telemonitor OR "tele-monitoring" OR "telemonitor" OR wireless OR wearable OR wearables OR sensor OR sensors OR sensing OR smart* OR IOT OR "signal processing" OR video OR camera OR "Inertial Measurement Unit" OR "Inertial Measurement Units" OR IMU OR "motion recognition" OR "real-time detection") OR ("Abstract":robot* or automation OR automated or technology or technological or technologies OR internet OR online OR computer OR computing OR AI OR "artificial intelligence" OR "machine learning" OR telemetry OR telemonitoring or telemonitor OR "tele-monitoring" OR "telemonitor" OR wireless OR wearable OR wearables OR sensor OR sensors OR sensing OR smart* OR IOT OR "signal processing" OR video OR camera OR "Inertial Measurement Unit" OR "Inertial Measurement Units" OR IMU OR "motion recognition" OR "real-time detection")) AND (("Document Title":compensation OR compensatory OR "non-use" OR "constraint induced movement" OR "constraint induced movements") OR ("Abstract":compensation OR compensatory OR "non-use" OR "constraint induced movement" OR "constraint induced movements"))

### Dissertations & Theses Global (Proquest)

noft(compensation OR compensatory OR "non-use" OR "constraint induced movement" OR "constraint induced movements") AND noft(robot* OR automat* OR technolog* OR internet OR online OR computer OR computing OR AI OR "artificial intelligence" OR "machine learning" OR telemetry OR telemonitor* OR "tele-monitoring" OR telemonitor OR wireless OR wearable OR wearables OR sensor OR sensors OR sensing OR smart* OR IOT OR "signal processing" OR video* OR camera* OR "Inertial Measurement Unit" OR "Inertial Measurement Units" OR IMU OR "motion recognition" OR "real-time detection") AND noft("upper limb" OR "upper limbs" OR "upper extremity" OR "upper extremities" OR "upper body" OR arm* OR shoulder* OR hand* OR axilla* OR elbow* OR forearm* OR finger* OR wrist* OR dexterity OR dexterous* OR grip OR gripping OR grasp OR grasping OR tap OR tapping OR reach OR reaching) AND noft(rehab* OR neurorehab* OR telerehab* OR physiotherap* OR "physio-therapy" OR "physio-therapist" OR "physical therapy" OR "physical therapist" OR Kinesiotherap* OR exercise* OR exercising) AND noft(stroke OR strokes OR poststroke OR poststrokes OR "post-stroke" OR "post-strokes" OR cerebrovasc* OR brain vasc* OR cerebral vasc* OR cva OR cvas OR apoplex* OR SAH OR SAHs OR TIA OR TIAs OR "Transient Ischemic Attack" OR "Transient Ischemic Attacks")

##

## Trials Registers

### ClinicalTrials.gov

Population: stroke OR strokes OR poststroke OR poststrokes OR "post-stroke" OR "post-strokes" OR cerebrovasc* OR brain vasc* OR cerebral vasc* OR cva OR cvas OR apoplex* OR SAH OR SAHs OR TIA OR TIAs OR "Transient Ischemic Attack" OR "Transient Ischemic Attacks"

Other Terms: compensation OR compensatory OR "non-use" OR "constraint induced movement" OR "constraint induced movements"

Limited to: Adult, Older Adult

### WHO ICTRP

Population: stroke OR strokes OR poststroke OR poststrokes OR "post-stroke" OR "post-strokes" OR cerebrovasc* OR brain vasc* OR cerebral vasc* OR cva OR cvas OR apoplex* OR SAH OR SAHs OR TIA OR TIAs OR "Transient Ischemic Attack" OR "Transient Ischemic Attacks"

Intervention: compensation OR compensatory OR "non-use" OR "constraint induced movement" OR "constraint induced movements"
